# Rat Models in Post-Traumatic Stress Disorder Research: Strengths, Limitations, and Implications for Translational Studies

**DOI:** 10.3390/pathophysiology31040051

**Published:** 2024-12-06

**Authors:** Alexey Sarapultsev, Maria Komelkova, Oleg Lookin, Sergey Khatsko, Evgenii Gusev, Alexander Trofimov, Tursonjan Tokay, Desheng Hu

**Affiliations:** 1 Institute of Immunology and Physiology, Ural Branch of the Russian Academy of Science, 106 Pervomaiskaya Street, 620049 Ekaterinburg, Russia; gusev36@mail.ru; 2 Russian–Chinese Education and Research Center of System Pathology, South Ural State University, 76 Lenin Prospekt, 454080 Chelyabinsk, Russia; mkomelkova@mail.ru; 3 National Scientific Medical Center, Astana 010000, Kazakhstan; lookinoleg@gmail.com; 4 Anatomical and Physiological Experimental Laboratory, Department of Experimental Biology and Biotechnology, Institute of Natural Sciences and Mathematics, 48 Kuybysheva Str., 620026 Ekaterinburg, Russia; hardscore@mail.ru; 5 Biology Department, School of Sciences and Humanities, Nazarbayev University, 53 Kabanbai Batyr Ave., Astana 010000, Kazakhstan; alexander.n.trofimov@gmail.com (A.T.); tursonjan.tokay@nu.edu.kz (T.T.); 6 Department of Integrated Traditional Chinese and Western Medicine, Union Hospital, Tongji Medical College, Huazhong University of Science and Technology, Wuhan 430000, China; desheng.hu@hust.edu.cn; 7 Hubei Key Laboratory of Biological Targeted Therapy, China-Russia Medical Research Center for Stress Immunology, Union Hospital, Tongji Medical College, Huazhong University of Science and Technology, Wuhan 430000, China

**Keywords:** ethological validity, model limitations, neuroendocrine factors, pharmacological interventions, PTSD, rats, rodent models, single prolonged stress (SPS), stress–re-stress paradigm, translational research

## Abstract

Post-Traumatic Stress Disorder (PTSD) is a multifaceted psychiatric disorder triggered by traumatic events, leading to prolonged psychological distress and varied symptoms. Rat models have been extensively used to explore the biological, behavioral, and neurochemical underpinnings of PTSD. This review critically examines the strengths and limitations of commonly used rat models, such as single prolonged stress (SPS), stress–re-stress (S-R), and predator-based paradigms, in replicating human PTSD pathology. While these models provide valuable insights into neuroendocrine responses, genetic predispositions, and potential therapeutic targets, they face challenges in capturing the full complexity of PTSD, particularly in terms of ethological relevance and translational validity. We assess the degree to which these models mimic the neurobiological and behavioral aspects of human PTSD, highlighting areas where they succeed and where they fall short. This review also discusses future directions in refining these models to improve their utility for translational research, aiming to bridge the gap between preclinical findings and clinical applications.

## 1. Introduction

Post-Traumatic Stress Disorder (PTSD) stands as a complex psychiatric condition marked by enduring psychological distress following exposure to a traumatic event. The quest to unravel the intricacies of PTSD has led to the development and utilization of various animal models aimed at simulating aspects of the disorder’s etiology, symptomatology, and treatment responsiveness. These models, including the single prolonged stress (SPS) model, stress–re-stress (S-R) paradigm, and the Footshock-Induced PTSD Model, among others, serve as critical tools in simulating the multifaceted nature of trauma exposure and PTSD symptomatology in animals. By replicating a multitude of aspects of the trauma and stress experienced by humans, these models provide invaluable insights into the pathophysiology of PTSD, uncover potential therapeutic targets, and facilitate the development of novel treatment strategies. The diverse approaches employed across these models, from exposure to stressors and fear conditioning to social and predator-based stress paradigms, reflect the ongoing effort to capture the essence of PTSD’s impact on the individual.

However, it is crucial to acknowledge the inherent limitations of translating animal behavior directly into human psychiatric conditions, steering clear of anthropomorphizing animal responses to stress. The complexity of PTSD, characterized by its diverse symptomatology and individual variability in response to trauma, poses significant challenges. As our knowledge expands through neuroscientific breakthroughs and omics-based analyses, the potential to refine these models and develop new ones grows. The present study delves into the current landscape of PTSD research through the lens of animal modeling, examining the progress made, the challenges encountered, and the horizons yet to be explored. This manuscript aims to bridge the gap between experimental research and clinical reality, shedding light on how far we have come and what remains to be discovered in the quest to understand and effectively treat PTSD.

PTSD models can be categorized into distinct groups reflecting their diverse strategies and research aims (Table 1). Specifically, the term “strategies” refers to the methodological approaches used in these models, the types of stressors employed (such as physical or psychological), and the targeted outcomes in terms of neurobiological mechanisms. These factors determine the classification and applicability of each model to different aspects of PTSD pathology.

Models using physical stressors such as footshock, stress-enhanced fear learning, restraint stress, and single prolonged stress (SPS) are widely accepted for studying PTSD. These paradigms are used alone or in combination to replicate varying degrees of physical stress and examine behavioral responses to subsequent stressors. Another kind of model involves predator-based stressors, which expose animals to predator stimuli like cat odor, fox odor, or trimethylthiazoline (TMT). These models aim to induce a sense of threat and helplessness, providing an ethologically valid experience that mimics natural environmental stressors. In addition to physical and predator-based stressors, social stressor models introduce critical dimensions of social interaction, such as social isolation, housing instability, and juvenile social exploration. These models emphasize the role of the social environment in modulating PTSD symptoms and the impact of temporal factors on the development and persistence of PTSD. All the models can be additionally classified as single-stressor and multi-stressor models, with short-term and long-term effects, with physical and non-physical treatment, and as mechanistic (genetic models, pharmacological treatments) or non-mechanistic (behavioral analysis) models.

Overall, understanding the nature of PTSD requires a comprehensive approach for categorizing animal models based on factors such as the type of stressor, temporal dynamics, complexity, and reproducibility of human responses to stress. In this review, we describe each classification type in detail, with extensive examples and relevant references to published studies. While the primary focus is on PTSD models developed in rats, mouse models are also discussed where they provide critical insights or unique advantages. For instance, the greater availability of genetically modified mouse models offers valuable opportunities to explore the genetic and molecular mechanisms underlying PTSD. Including these references enriches this review by presenting complementary findings from both species.

## 2. Single-Stressor Models

### 2.1. The Electric Shock Models

**Figure 1:**
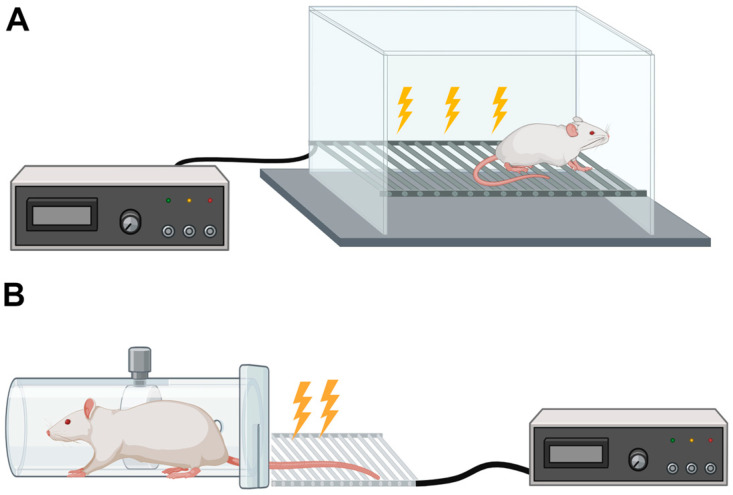
Schematic picture of two classical designs for the electric shock models—Footshock (**A**) and Inescapable Tail Shock models (**B**). The animal is kept inside the chamber to restrict movement during shocks. The electrical impulses are supplied either on the floor to affect the feet (**A**) or to the panel to which the tail can touch.

The electric shock models, including the Footshock-Induced PTSD Model and the Inescapable Tail Shock (ITS), offer valuable frameworks for understanding PTSD by exploring the mechanisms of fear conditioning and stress induction. These models utilize electric shocks, delivered to either the animal’s tail or foot, to simulate traumatic events, thereby linking the onset of PTSD-like symptoms to the processes underlying fear responses. This connection is crucial because fear and associated behaviors such as hyperarousal, avoidance, and exaggerated startle responses are central components of PTSD [1,2,3]. Figure 1A,B schematically show two classical designs for the electric shock models—the Footshock and Inescapable Tail Shock models, respectively.

Electric footshock stress incorporates both physical and psychological stressors. However, habituation to these stressors can vary significantly depending on the animal strain and context [4]. For instance, studies have shown that while some strains may not habituate to footshocks, others might, especially when the intensity and frequency of the shocks are controlled [5]. This variability challenges the assumption that electric shocks are inherently non-habituating and underscores the importance of careful experimental design.

The etiological validity of electric shock models lies in their ability to simulate the unpredictable, intense nature of traumatic events akin to human experiences. However, variability in responses among animals is a critical consideration. While some models demonstrate uniform stress-related behavioral outcomes, this uniformity might not accurately reflect the diverse human responses to trauma. High variability in human PTSD responses suggests that animal models should ideally capture a range of stress responses to better model the disorder’s complexity.

Electric shock models enable detailed analysis of fear circuitry, revealing changes in neural activation, morphology, and signaling within key brain regions such as the prefrontal cortex, amygdala, and hippocampus. These models support construct, predictive, and face validity for PTSD research by revealing alterations in neuroendocrine functions and neuroplastic changes [6,7]. Furthermore, the combination of electric shocks with other stressors, such as restraint or corticosterone injection, provides deeper insights into the neuropsychological and molecular underpinnings of PTSD [8,9]. For instance, combining footshock with social isolation has been shown to exacerbate PTSD-like symptoms, offering insights into the interaction between social and physical stressors [10,11,12,13]. These combined models effectively induce hyperarousal, avoidance behaviors, and impaired fear extinction—hallmark symptoms of PTSD. Berardi et al. (2014) showed that social isolation following electric footshock is a remarkable risk factor for developing the anxious behavioral profile in the social interaction test and reducing locomotor activity in the elevated plus maze test, mirroring core features of PTSD pathology in humans [11]. Morena et al. (2018) demonstrated that pharmacological interventions targeting the endocannabinoid system during extinction learning could mitigate these effects, suggesting an avenue for enhanced treatment efficacy [10].

Various adaptations of this model include protocols that subject rodents to intense electric footshocks followed by situational reminders to reactivate trauma memory [14]. For example, Louvart et al. refined the model, showing long-lasting PTSD behaviors after a single 2 mA electric shock and subsequent weekly reminders [9,15]. The stress-enhanced fear learning (SEFL) paradigm extends this concept by demonstrating how subsequent shocks in new environments amplify the fear response, enriching our comprehension of PTSD’s enduring effects [8,16]. SEFL is particularly effective for assessing learned fear and fear memory in animals, providing long-term insights into PTSD-like symptoms.

It is essential to distinguish between paradigms that use electric shock to induce long-term PTSD-like symptoms and those that employ shocks for fear conditioning. In PTSD models, shocks are typically of higher intensity (1.0 to 3.0 mA) and duration (2 to 20 s), intended to simulate the severe impact of trauma, including unpredictable timing to enhance the stressor’s unpredictability. In contrast, fear conditioning models use lower amplitude shocks (0.5 to 1.0 mA) and shorter duration (less than 2 s) to study basic mechanisms of fear acquisition, extinction, and reinstatement, focusing on conditioned learning rather than inducing a traumatic state [17,18].

There are two significant advantages of simple conditioned fear models in PTSD research: high face validity—as a single shock session can induce a robust fear response with strong associative memory—and changes in fear conditioning, such as increased fear acquisition, impaired extinction, and a tendency for relapse [18]. Despite their feasibility, these models face limitations such as the artificial setting of electric shocks, which may not fully replicate human PTSD experiences [17,18,19,20,21,22]. Findings by Viellard et al. (2024) highlight limitations in traditional shock-based models, particularly regarding the importance of threat avoidance. Models that allow animals to dynamically avoid threats show activation of neural circuits closely resembling responses to more naturalistic threats, emphasizing the importance of adaptive responses in understanding PTSD [23].

A key limitation is the inability of single-stress models to capture the dynamic nature of real-world threat detection and avoidance, crucial for understanding PTSD’s complexity. Ethical concerns also arise due to the potential physical harm to animals from high-intensity shocks, alongside inconsistencies in replicating specific PTSD markers, such as altered hypothalamic–pituitary–adrenal (HPA) axis function and corticosterone levels, particularly in low-intensity shocks [21,24,25]. Selecting the appropriate model must balance uniformity and variability in stress responses to meet specific research goals effectively.

### 2.2. Immobilization Stress Models

**Figure 2:**
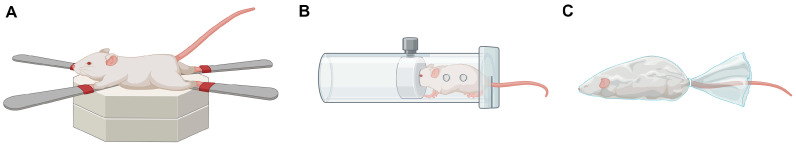
Schematic picture of different approaches used to restrict the movement of an animal. (**A**) Each foot of the animal is fixed to prevent movement while the body is not fixed (the animal can move its head and tail). (**B**) The animal is placed in a transparent chamber restricting its lateral movement to the desired extent (adjusted by the restriction block). (**C**) The animal is fully enclosed by a transparent bag restricting its movement while not preventing its breathing. Note that all approaches do not block seeing or hearing, thus simulating stress in humans.

In stress research, the terms “restraint” and “immobilization” stress (IS) are often used interchangeably, but they refer to distinct procedures with critical differences that influence the outcomes of experiments. As delineated by Armario et al. (2004), both restraint and IS protocols restrict movement, but they differ in the degree of motion limitation imposed on the subject [26]. Restraint partially limits the movement of the rodent’s limbs, body, and head, whereas IS imposes more severe restrictions, often completely preventing locomotion. This is typically achieved by confining the animal in an immobilization bag, such as a Decapicone, or securing it to a board in a fixed position (Figure 2).

IS models are often perceived to elicit stronger physiological and behavioral responses compared to restraint. By completely restricting movement and potentially combining IS with other stressors, these models simulate severe and unpredictable conditions to replicate the complexity of trauma that may lead to PTSD-like symptoms, thereby enhancing their etiological relevance [25,27,28,29,30,31]. However, recent evidence suggests that the outcomes of both IS and extended restraint can be quite similar, highlighting the importance of experimental designs like the duration and specific conditions of stress application in maintaining this validity [24,32,33,34].

IS models have strong construct validity, evidenced by their ability to produce changes at the molecular and cellular levels that align with known PTSD pathology. These include increased negative feedback within the HPA axis, heightened behavioral anxiety, and nociception, paralleling symptoms observed in PTSD patients. The use of IS has further shown spatial memory impairments, increased spine density, and enhanced long-term potentiation) in brain areas linked to PTSD, such as the hippocampus [35,36,37]. Additionally, immobilization on boards, as studied by Sanz García et al. (2016), induces long-term behavioral effects (2–8 days) akin to those seen in PTSD patients [37].

It is important to interpret the results from IS models with caution. The study by Louvart et al. (2006) involved a complex methodology, including electric shocks followed by weekly situational reminders and a final restraint stress 41 days later [9]. The observed differences in corticosterone levels in female rats were specific to a 60 min time point and may have reflected a delayed response to the initial electric shock rather than a direct effect of restraint stress. Additionally, the study found no significant differences in the expression of glucocorticoid and mineralocorticoid receptors in different regions of the hippocampus between shocked and non-shocked females, although there was a difference in males in the CA2 region of the hippocampus. This suggests that the observed effects cannot be solely attributed to restraint stress and raises questions about whether they represent a sex-specific response to IS or a late effect of the prior electric shock. Therefore, it is not accurate to conclude that only female rats respond to this stress model, and this should not be considered a limitation of IS. Other studies, such as Barha et al. (2011), have shown sex differences in stress responses after chronic restraint stress, indicating that these differences are a broader characteristic of stress responses rather than a limitation specific to the IS model [38].

IS models have notable strengths, including extensive data on HPA axis-related changes and time courses, as well as detailed analysis of structural and functional changes in the prefrontal–hippocampal–amygdala network. The availability of data on both sexes and the reliable effects on fear-specific processes further enhance the model’s utility in PTSD research [22]. The current state of studies using IS models may reflect gaps in research, such as limited data on other PTSD-relevant behavioral or biological outcomes, rather than inherent limitations of the model itself. The limited application of vulnerable-resilient subgrouping approaches in IS models [22] should be seen as a promising avenue for future research rather than a limitation of the model. Expanding this approach could enhance the model’s utility by providing deeper insights into individual differences in stress responses. In addition, the IS protocols can be combined with other stressors, such as exposure to predator scent, social defeat, or electric shock, to amplify stress responses and create more complex PTSD-like conditions [25,26,27,28,29,30]. This further enhances the model’s ability to mimic the multifaceted nature of PTSD pathology as best as possible.

Overall, while IS models, including both restraint and more severe IS protocols, offer valuable insights into the (patho)physiological and behavioral responses associated with PTSD, they also face challenges. These include issues related to the naturalistic relevance of extreme immobilization and variability in stress responses across sexes. Such considerations should guide future research to enhance the applicability and generalizability of findings from these models [22].

### 2.3. The Underwater Trauma (UWT) and Water Avoidance Stress (WAS) Models

**Figure 3:**
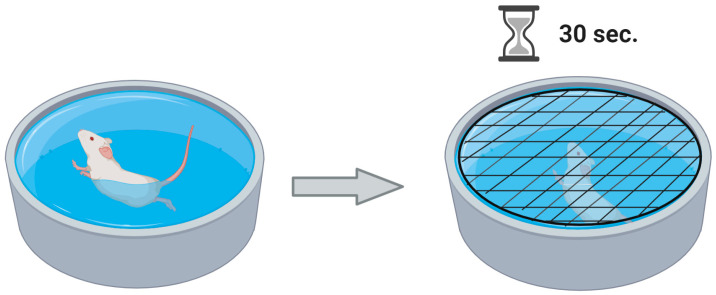
A simplified representation of the underwater trauma (UWT) model used to stress an animal. The animal is allowed to swim in an open pool without an underwater platform for a short period (typically 30 s). The pool is closed by a metal net to submerge the animal underwater and induce sudden stress because the animal cannot emerge from the water during this time.

The underwater trauma (UWT) and the Water Avoidance Stress (WAS) models serve distinct but complementary purposes within PTSD and behavioral research. The UWT model is mainly utilized to induce stress and is designed to simulate a life-threatening situation that evokes PTSD-like symptoms in rodents. In the UWT model described by Richter-Levin in 1998, both UWT and aspects of the Water-Associated Zero Maze (WAZM) test were employed to explore behavioral and pathophysiological symptoms indicative of PTSD in rats, with a strong emphasis on the role of contextual elements in the manifestation of post-trauma symptoms [39]. In this UWT model, rats swim in a maze for up to one minute without a platform before being submerged underwater for 30 s using a special metal net, after which they are placed in a resting cage until post-trauma tests begin (Figure 3). Control rats swim for one minute and are then moved to a resting cage without undergoing underwater trauma. The underwater trauma results in acute and lasting behavioral effects, including poor performance in spatial memory tasks within the water maze and avoidance of the open arms of the elevated plus maze both an hour and three weeks post-trauma, suggesting that the observed deficits are not exclusively related to spatial memory but may involve other cognitive or attentional processes as well [39].

Additionally, stressed rats exhibit decreased plasma basal corticosterone levels seven days post-stress [40], indicating prolonged HPA axis depression, and increased anxiety-like behavior [41]. These physiological outcomes contribute to the construct validity of the UWT model by linking changes at the molecular level, such as HPA axis dysregulation, to PTSD symptoms observed in human patients. Studies, such as those by Ardi et al. (2014), have demonstrated that exposure to UWT reminders further exacerbates anxiety behaviors and impairs memory-related mechanisms in the dentate gyrus and the amygdala, emphasizing their role in maintaining PTSD-like symptoms [40,41,42,43,44,45].

The choice of UWT as a stressor is particularly relevant due to its life-threatening nature, brief duration, and capacity to allow for subsequent assessments of trauma effects on memory and attention within or outside the trauma’s context. This demonstrates high ethological validity as it offers a more “natural” stress setting compared to electric footshocks/tail shocks. Importantly, this model does not inflict noticeable physical harm, which could otherwise explain the poor performance, thus underscoring its utility in simulating psychological and contextual stress factors [39]. The etiological validity of the UWT model is highlighted by its ability to simulate a life-threatening experience that is directly related to the development of PTSD-like symptoms. The model’s ability to evoke both immediate and long-term responses mimics human trauma, contributing to its etiological relevance [40].

A critical distinction in UWT models is between the initial trauma exposure and subsequent reminder exposures. The initial underwater trauma serves to induce long-term PTSD-like symptoms, simulating a life-threatening experience that leads to persistent changes in both behavior and neurophysiology. In contrast, trauma reminders, such as re-exposure to the underwater context, are used to replicate the re-experiencing of trauma—a key feature of PTSD pathology. This distinction highlights how both initial trauma and reminders serve different but complementary roles in understanding the mechanisms underlying PTSD. Moreover, this suggests that re-experiencing elements may be crucial for understanding both the behavioral and neurobiological persistence of PTSD-like symptoms. Behavioral profiling refined through multiple behavioral tests, as seen in recent studies, is also crucial for identifying PTSD-like versus resilient phenotypes [43].

In contrast to the UWT model, the WAS model involves exposing rodents to a stressful environment where they must avoid water by balancing on a small platform, inducing stress responses without a direct threat to life [46,47]. The WAS model may have modifications in the context of how the stress is prolonged (i.e., single or repeated WAS) or whether the stressor can be “controlled” by the animals or not. For water avoidance purposes, this means either the presence or absence of the platform to avoid the stressor. This aspect demonstrates moderate ethological validity, as the WAS model creates a challenging situation without the immediacy of life-threatening danger. Repeated WAS trains applied to rats for several (typically 3 to 10) consecutive days induce not only behavioral changes but also physiological alterations in visceral function/sensation like visceral pain and mucosal immune activation, which are thought to be mediated by activation of corticotropin-releasing factor (CRF) [46,47,48,49,50,51]. These physiological changes contribute to the construct validity of the WAS model by revealing systemic physiological responses to stress that align with PTSD pathology.

The WAS model is effective at producing distinct stress responses, such as heightened anxiety and increased avoidance behaviors, but without directly threatening the animal’s life. This reflects behavioral outcomes that are useful for modeling PTSD symptoms, though its generalizability is limited when trying to simulate purely psychological trauma without physiological confounding factors.

The Morris water maze test (MWMT) and Water-Associated Zero Maze (WAZM) are used to assess cognitive functions such as spatial memory and learning after water-associated stress. These tools act as diagnostic rather than stress-inducing models, helping to evaluate the long-term cognitive impact of traumatic stressors like UWT and WAS. The MWMT is critical for examining the impact of stress on hippocampal-dependent cognitive processes, providing insights into the mechanisms of cognitive dysfunction in PTSD. The WAZM has been proposed for inducing traumatic memory re-experiencing, which can be instrumental in understanding the neural underpinnings of PTSD [52,53].

In summary, the UWT model’s strengths lie in its ability to simulate naturalistic, life-threatening stressors without inflicting noticeable physical harm, allowing for the assessment of both immediate and long-term behavioral and (patho)physiological responses. It demonstrates lasting changes in anxiety-like behavior and HPA axis activity [39,40,41,42,43,44,45,46,47,48,49,50,51,52,53]. However, using this model in rats has uncovered that animals exhibit at least three different phenotypes to the stress: resilient, anxiety–fear-based, and comorbid fear–anhedonic [54]. This highlights the importance of individual susceptibility to stress and suggests that individual behavioral profiling of animals is essential for the correct interpretation of the neural basis of PTSD [55,56].

### 2.4. Sudden Sound Stress and Acoustic Startle Response

Sound, especially sudden and loud, is a substantial contributor to the constantly increased number of noise-related neurological and psychological disorders [57]. As a potent stressor, loud sound (alone or in combination with other stressors) is extensively used in human studies, many of which concern PTSD-related issues like elevated vulnerability and diminished habituation to stress [58,59,60]. For example, in military blast explosion survivors, increased sound intolerance has been reported and associated with an increased prevalence of PTSD [61]. Also, sudden sound-induced temporal occurrence of rest, postural, and kinetic tremor has been documented, and it was linked to a psychogenic origin similar to the development of PTSD [62]. The brief but high-intensity sound elicits neurological reactions that are distinct or enhanced in PTSD patients and PTSD-like animals [63]. In experimental studies on animals, sudden loud sound is a powerful stressor triggering behavioral and neurological alterations, particularly in the context of PTSD-like states [64,65,66], and is widely used to induce neural activation in susceptible and hypersensitive animals, like epilepsy-prone rodents [64,67,68,69]. In rodents, this approach is helpful in studying the links between audiogenic stress and subsequent modifications and adaptations in central nervous circuits to reproduce typically observed human responses to stressful environments [70,71,72,73,74,75,76,77,78,79,80].

The Sudden Sound Stress (SSS) model has high ethological validity due to its use of natural, salient stimuli—loud sounds—that mimic real-life sudden stressors like explosions or gunfire, which are common sources of trauma in humans. The unpredictability and intensity of the sound contribute to the high ecological relevance of this model, making it effective for simulating PTSD-like hyperarousal responses in rodents.

The etiological validity of the SSS model lies in its ability to simulate sudden, intense auditory stress similar to the unexpected sounds that can serve as traumatic events in human PTSD. This unpredictability aligns well with the nature of many traumatic experiences, making the SSS model particularly effective for replicating the types of triggers that can lead to PTSD in humans. The acoustic startle response (ASR) is a crucial element in PTSD research, allowing for the assessment of anxiety and hyperarousal as well as the alterations in neural circuits related to fear and anxiety, thereby validating its use as an important tool for studying the disorder’s underlying mechanisms. By using ASR for phenotyping, researchers can separate rats or mice into different groups based on the strength of their startle response to identify animals that are either susceptible or resilient to PTSD-like symptoms [71,72,73,74].

The construct validity of models utilizing ASR is supported by its capacity to reflect neurophysiological changes associated with PTSD. The ASR is used to measure the reflexive startle reaction in rodents, which is often heightened following traumatic experiences. Studies have shown that exposure to SSS results in altered neurotransmitter levels, including serotonin and norepinephrine, and increased activity in brain regions like the amygdala and prefrontal cortex—changes that are consistent with those observed in PTSD patients. Neuroendocrine studies suggest a link between modifications in the startle response and dissociative states, with heightened cortisol or corticosterone levels implicated in the attenuation of startle reactions post-extreme stress [76,77,78]. Additionally, baseline ASR levels may predict PTSD-like symptoms, as rats displaying a lower baseline ASR tend to show diminished retention of extinction memory [73]. These molecular and physiological changes underline the strong construct validity of the ASR-utilizing model. The ASR’s sensitivity to changes in neural circuits makes it a valuable tool for screening individuals for trauma susceptibility and resilience under specific disorders. Moreover, this screening can be complemented with molecular measures, such as assessing changes in neuroendocrine markers like corticosterone levels or evaluating molecular and genetic markers associated with stress response pathways.

Both the SSS and ASR models induce distinct behavioral outcomes that are indicative of PTSD-like symptoms. Animals exposed to SSS often exhibit increased anxiety, hypervigilance, and heightened startle responses, all of which are characteristic of PTSD. The ASR provides a quantitative measure of hyperarousal by tracking the amplitude and latency of the startle reflex, which serves as an indicator of the severity of the PTSD-like state. This allows researchers to assess not only the immediate effects of acute sound stress but also how repeated exposures contribute to the persistence of PTSD-like symptoms over time. Findings also indicate that changes in the startle reaction shortly after trauma—particularly within the first hour—are more predictive of PTSD onset than alterations observed 24 h later [79]. Additionally, baseline ASR levels may predict PTSD-like symptoms, as rats displaying a lower baseline ASR tend to show diminished retention of extinction memory [73]. Moreover, while some studies have shown that startle magnitude increases following mild footshocks, other research suggests that severe stress might further attenuate the startle response, although the evidence is mixed and requires further investigation [79,80].

Overall, the SSS and ASR models are valuable tools in PTSD research due to their high ethological and construct validity. The use of sudden, loud sounds as stressors effectively mimics the types of traumas that lead to PTSD in humans, and the ASR provides a reliable measure of hyperarousal. However, it is important to note that the treatment with sudden sound is primarily a single-stressor model rather than a multi-stressor or ethologically valid model. The use of loud sounds as a stressor does not fully replicate the natural stressors that animals might encounter in the wild. In the context of PTSD research, while the ASR can be used as a screening tool to evaluate the severity of PTSD-like symptoms or as part of a diagnostic protocol, its limitations in ecological validity should be considered, particularly when assessing its role in simulating the full spectrum of PTSD symptoms. Employing sudden sound and measuring the ASR within broader screening frameworks, such as the arousal-based individual screening (AIS) model, enhances its application [81]. The AIS model subjects rodents to trauma and implements individual screening methods to identify those with enduring PTSD-like phenotypes and those exhibiting resilience through active coping mechanisms.

## 3. Intermediate Complexity Models

### 3.1. Single Prolonged Stress (SPS)

Developed by Liberzon et al. (1997, 1999), the SPS model involves a single prolonged stress session that induces increased negative HPA axis feedback, a consistent neuroendocrinological characteristic of PTSD [82,83]. The model combines a series of stressors—two hours of restraint and a 20 min forced swim, followed by diethyl ether anesthesia—to replicate the intricate interplay of physical and psychological stressors pivotal in the onset of PTSD [84,85,86,87]. Although some studies have incorporated a period of social isolation following the SPS session, this is not a standard component of the model. According to a review by Ferland-Beckham et al. (2021), only 12% of SPS studies used social isolation, and the results were consistent regardless of its inclusion in the session [85,87]. This suggests that social isolation may not be a necessary component for achieving the model’s outcomes. The strength of the SPS model lies in its ability to synergistically combine these stressors, closely mimicking the complex nature of trauma exposure [85]. This approach has been crucial in uncovering neurobiological changes associated with trauma, including alterations in the oxytocin system, which is vital for stress regulation and social behaviors. Studies, notably by Liberzon and Young (1997), have highlighted the hormonal and neurobiological shifts following trauma, thus enriching our understanding of PTSD’s underlying mechanisms [88,89].

A distinctive feature of the SPS model is its incorporation of a 7-day or 14-day undisturbed sensitization phase following stressor exposure. This period is intended to allow the animal to process and “memorize” the traumatic events, facilitating the development of PTSD-like symptoms. However, the idea that most behavioral and cellular alterations only become evident after this latency period is more speculative than definitive. While some studies, such as those by Wu et al. (2016) [90], observed more pronounced behavioral changes on day 7 compared to day 1, other studies, like the one by Liberzon et al. (1999) [83], have reported cellular changes, such as decreased hippocampal glucocorticoid receptor expression, as early as day 1 post-stress. This suggests that while the sensitization period may be important, the timing of observable changes can vary depending on the specific type of alteration being measured. Also, this delay in the behavioral effects relative to the observed cellular changes can simply reflect the time frame needed for the completion of structural and functional neuronal changes triggered by the stressors. The 7-day latency should be considered as a recommended interval rather than a strict time frame, particularly when differentiating between behavioral and cellular responses.

The use of multiple stressors in the SPS model raises concerns regarding its ethological relevance. The combined stressors—restraint, forced swim, and ether anesthesia—are far from replicating naturalistic traumatic events that animals might encounter in the wild. These stressors are artificial and may not accurately reflect the trauma environment faced by humans, thus limiting the ecological validity of the model. However, despite these concerns, the SPS model has been effective in eliciting PTSD-like symptoms, particularly related to stress-induced hormonal changes, altered social behaviors, and persistent anxiety responses [84,85,86,87]. This model is effective in simulating the synergistic nature of cumulative trauma, which reflects the human experience of enduring complex stress exposure.

The SPS model provides strong etiological validity by simulating the cumulative and complex nature of traumatic experiences, which often involve multiple stressors [83,84,85,86,87,88,89,90,91,92,93,94,95,96,97,98,99,100]. The combination of physical (e.g., restraint and forced swimming) and psychological stress (e.g., unpredictable ether anesthesia) mimics the intricate interplay of human PTSD, capturing both physical adversity and emotional trauma. Additionally, the incorporation of a sensitization period (7 or 14 days) following the initial stress exposure aims to reflect the delayed onset of PTSD symptoms. This sensitization period facilitates the development of PTSD-like symptoms by allowing time for consolidation of the trauma memory and subsequent behavioral manifestations [83,90]. However, some studies have observed variations in the timing of behavioral and cellular changes—indicating that the latency phase should be seen as a recommendation rather than a strict requirement for consistent outcomes [83,90].

The SPS model also demonstrates considerable construct validity through its ability to induce neurobiological changes that align with PTSD pathophysiology. Studies have observed alterations in the oxytocin system, decreased hippocampal glucocorticoid receptor expression, and increased inhibition of HPA axis negative feedback following SPS exposure [88,89,99,100]. These neuroendocrine shifts are consistent with PTSD, providing insights into stress regulation and related behavioral changes. Furthermore, increased basal plasma glucocorticoid levels have been observed in some instances following SPS, a characteristic commonly linked to psychiatric conditions like depression and anxiety, although its relationship to PTSD remains complex due to variability across studies [94,95,96,97,98]. Moreover, the use of ether as a component of the SPS protocol is particularly noteworthy because of its effects on membrane permeability and potential neurotoxicity, possibly acting through mechanisms of anoxia, which may contribute to PTSD-like phenotypes [22]. Recent alternatives to using ether, such as substituting it with social stress, have yielded similar physiological outcomes, further validating the model’s construct [101,102,103].

The behavioral outcomes induced by the SPS model align closely with PTSD, including heightened anxiety, avoidance behaviors, and impaired fear extinction retention. Some studies have questioned its ability to consistently elicit trauma-specific avoidance behaviors; however, other research, such as that by Ganon-Elazar and Akirav (2012), has demonstrated increased avoidance of trauma-related stimuli, such as electric shock cues [91]. These findings indicate that the SPS model can indeed reproduce habit-like avoidance responses to trauma, which is critical for understanding PTSD’s behavioral aspects. Notably, the incorporation of a sensitization phase allows for a more realistic examination of PTSD-like symptoms, including prolonged anxiety responses and difficulties in extinguishing conditioned fear [90,91].

A promising advancement in stress modeling is the Multiple Prolonged Stress (MPS) model, which builds upon the SPS framework by incorporating repeated and randomized exposures to stressors over a seven-day period [104]. Unlike the single-event nature of SPS, MPS emphasizes prolonged and cumulative stress, allowing for enhanced simulation of chronic trauma. This modification has been shown to result in more robust and sustained PTSD-like symptoms, including extended fear memory retention, persistent anxiety-like behaviors, and heightened neuronal activity in critical brain regions such as the prefrontal cortex, amygdala, and hippocampus [104]. Additionally, it induces prolonged dysregulation of the HPA axis and significant disruptions to sleep patterns, including reduced rapid eye movement (REM) sleep and increased slow-wave activity, which are associated with chronic stress adaptation and fear memory consolidation. These enhancements make the MPS model a promising tool for capturing the complexity of PTSD and for developing therapeutic interventions [104].

The SPS model has highlighted profound sex differences in both physiological and behavioral responses to trauma, which bear significant implications for the generalizability of PTSD research [105,106,107]. The findings from studies like those by Keller et al. (2015) indicate that SPS reduces fear extinction deficits primarily in males, suggesting variability in stress vulnerability across sexes [102]. Male rats frequently show heightened hyperarousal, impaired fear extinction, and increased dopaminergic activity following SPS exposure. In contrast, females tend to exhibit depressive-like responses, reduced glucocorticoid receptor sensitivity, and greater activation in stress-related brain regions such as the prefrontal cortex and amygdala [106,107,108]. Pooley et al. (2018) reported that female rats, particularly those gonadectomized, displayed depressive tendencies akin to the internalizing symptoms seen in female PTSD patients, whereas males showed more externalizing symptoms [106]. Furthermore, studies have shown significant differences in fear extinction, with male rats displaying impaired extinction retention and females showing increased resilience, indicating that PTSD vulnerability and resilience mechanisms are sex-specific [102,108]. Molecular analyses have also shown differential glucocorticoid receptor expression, cFos activation, and endocannabinoid signaling between sexes, highlighting unique neurobiological responses in males and females [107,109]. Recent studies also emphasize divergences in behavioral and physiological outcomes; for example, males exhibited significantly heightened ASR following SPS exposure, while females did not show such changes, pointing to inherent differences in stress responsivity [107,108]. SPS resulted in a significantly higher anxiety index in male vs. female rats [110] and induced long-term emotional alterations only in male rats, i.e., up to 30 days following trauma exposure [108]. Of interest, recent studies revealed that the gut microbiome can be involved in the regulation of the stress response in a sex-specific manner, e.g., Cyanobacteria may be associated with anti-anxiety effects observed in male rats [111]. Furthermore, SPS-induced reductions in locomotor activity and altered novelty-seeking behaviors were noted in both sexes, with distinct underlying dopaminergic mechanisms observed—such as increased basal dopamine levels in the nucleus accumbens in males, which was not seen in females [108,110,112,113].

Fear extinction also demonstrated considerable sex-specific differences, with male rats exhibiting impaired fear extinction retention and females displaying increased extinction recall, suggesting divergent fear memory processes. Additionally, acetylcholinesterase activity during fear extinction showed greater impairment in females, alongside higher enzymatic activity in the amygdala, potentially contributing to greater PTSD vulnerability in females [114]. Differences were also observed in glucocorticoid receptor sensitivity, cFos expression, and the regulation of the endocannabinoid system, pointing to sex-specific limitations in therapeutic efficacy for eCB signaling enhancement [106,109].

The reproducibility of studies employing the SPS model is often undermined by significant methodological challenges that include variability in the scoring methods for behaviors such as freezing, the absence of randomization, and the lack of proper sample size calculations [87,105]. Such limitations are not unique to the SPS model but are common across many preclinical PTSD models, contributing to inconsistent outcomes and hindering the reliability of findings. This broader issue with reproducibility highlights a critical need for more rigorous standardization across PTSD research involving animal models [87,105]. For example, variability in the conditions of animal housing, the handling of subjects, and the tools used for behavioral assessments can lead to substantial differences in observed outcomes, affecting not only SPS but also other PTSD-like models.

In summary, the SPS model offers a comprehensive framework for investigating PTSD through a combination of stressors [22]. These findings align with known neuroendocrine indicators of PTSD, providing insights into the disorder’s pathophysiology, including psychological hyperarousal as defined by the DSM-5 [86]. Additionally, the model has revealed associations between fear extinction as well as increased anxiety-like behaviors and increased expression of glucocorticoid receptors in the hippocampus and prefrontal cortex, highlighting potential therapeutic targets [84,85,86,89]. Nevertheless, the limitations of the SPS model, such as its insufficient incorporation of learning processes, inconsistent reproducibility, and difficulties in regulating the intensity of stressors, underscore the need for models that combine both stress exposure and learning components to more accurately capture the full range of PTSD characteristics [115].

### 3.2. Unpredictable Variable Stress (UVS)

The Unpredictable Variable Stress (UVS) model represents a distinct approach to studying chronic and unpredictable stress exposure, differentiating it from the single prolonged stress (SPS) model. While SPS models the acute effects of trauma followed by delayed PTSD-like symptoms, UVS extends the scope by focusing on the chronicity and variability in stress exposure, making it well-suited for exploring resilience and susceptibility to PTSD [116]. UVS involves daily exposure to diverse and unpredictable stressors, such as restraint, noise, cold, and forced swimming, over a period of weeks. This chronic and uncontrollable stress mirrors the unpredictability of real-life trauma.

Although traditionally associated with depression research, UVS also demonstrates significant relevance to PTSD. It exhibits both face and predictive validity for certain PTSD-like symptoms by inducing behavioral phenotypes consistent with the disorder [22]. Moreover, UVS can be integrated into more complex models. For example, Wakizono et al. (2007) demonstrated that preceding UVS enhanced hyperactive behavioral changes in Wistar rats after inescapable electric footshocks, mimicking characteristic features of PTSD [116]. Similarly, UVS rats subjected to contextual fear conditioning and extinction tests, and allowed to recover for one week, displayed significantly increased freezing responses to trauma reminders compared to non-stressed rats. This behavior suggests enhanced fear recall, a hallmark of PTSD [116].

The UVS model has several strengths and limitations that define its utility in PTSD research. It demonstrates face validity by simulating behavioral and neurobiological features of PTSD, such as heightened anxiety and HPA axis dysregulation, aligning with observed human symptoms. Additionally, UVS exhibits predictive validity, as its responsiveness to pharmacological treatments such as selective serotonin reuptake inhibitors (SSRIs) and ketamine [117,118] mirrors clinical outcomes in humans, supporting its relevance for evaluating therapeutic interventions. The model also achieves construct validity by inducing chronic and unpredictable stress, which leads to increased corticosterone levels and changes in neurotransmitter systems, such as serotonin and dopamine, reflecting the core mechanisms of PTSD.

Despite these strengths, the UVS model has limitations that must be addressed. Reproducibility issues arise from variability in protocol implementation, including differences in timing and the sequence of stressors, which can lead to inconsistent results across studies. The reliance on subjective interpretations of behavioral outcomes further exacerbates this inconsistency. Additionally, the artificial stressors used, such as restraint or cold exposure, lack direct naturalistic relevance, which limits the ecological validity of the model. Chronic stressors may also fail to replicate the acute and variable nature of human trauma, and repeated exposure can result in habituation or desensitization, diminishing the ability to sustain PTSD-like states. Furthermore, differences in stress susceptibility across animal strains, sex, and age are not consistently accounted for, reducing the generalizability of findings. Nevertheless, some behavioral features have revealed their strong correlation to the outcomes of UVS in rats, e.g., hyper-responsivity to novelty and mild threats as well as the response typical for high anxiety/depressive animals [119]; therefore, they can be used as predictors of the extent of stress-induced changes. Ethical concerns regarding stress-inducing methods, such as restraint or forced swimming, further limit the applicability of the UVS model and may require refinements [120].

In summary, while the UVS model may not be exclusively classified as a PTSD model, it complements models like SPS by addressing the chronic and variable aspects of stress exposure. This makes it a valuable tool for studying PTSD-like behaviors, particularly in understanding resilience, susceptibility, and responses to therapeutic interventions.

### 3.3. The Stress–Re-Stress (S-R) and Differential Contextual Odor Conditioning (DCOC) Paradigms

The stress–re-stress (S-R) paradigm, as delineated by Liberzon, Krstov, and Young in their seminal 1997 study, introduces a methodological approach to modeling PTSD in animals by inducing an anxiety-like state [82]. This paradigm seeks to replicate the intricate cycle of experiencing a traumatic event followed by subsequent re-exposure to stimuli reminiscent of the initial trauma, effectively simulating the trigger–response mechanism characteristic of PTSD.

Within the S-R model, animals are subjected to a carefully designed sequence of stress-inducing procedures, beginning with two hours of confinement in a Plexiglas restrainer to elicit a psychological stress response through immobilization. This is followed by a forced swim test in ambient water for twenty minutes, intensifying the stress response through physical challenge and survival instinct activation. A 15 min recovery period precedes exposure to ether vapors, inducing unconsciousness and introducing an unexpected and uncontrollable factor to simulate the disorienting and unpredictable nature of traumatic experiences.

The etiological validity of the S-R model lies in its structured re-exposure to stress. After the initial stressors, animals are returned to their cages for a six-day period of undisturbed incubation, allowing PTSD-like symptoms to develop. The paradigm concludes with a re-stress session involving additional restraint stress, critical for reactivating traumatic memories and evaluating the persistence of stress-induced (patho)physiological and behavioral changes. However, Viellard et al. (2024) caution that traditional re-stress methods, such as electric shock, may not fully engage the threat detection and avoidance circuits relevant to PTSD [23]. Incorporating opportunities for animals to detect and avoid threats during re-stress sessions could enhance the model’s relevance by better simulating the anticipatory and avoidance behaviors seen in PTSD.

Further developments of the S-R paradigm have sought to address its limitations in construct validity. Guo et al. (2018) introduced intense footshock (FS) as an alternative re-stress cue, observing that FS provoked long-lasting anxiety and depression-like behaviors weeks after initial exposure, demonstrating a broader array of PTSD-like symptoms [121]. Unlike the traditional forced swim test (FST), FS-induced models also evoke hyperarousal and intrusive memory symptoms, with paroxetine treatment notably mitigating these behavioral deficits [122]. The significant neuroendocrine alterations associated with PTSD, such as marked changes in corticosterone levels and enhanced negative glucocorticoid feedback, suggest both adaptive mechanisms and potential maladaptations within the HPA axis. These findings underscore the S-R model’s value in elucidating the neurobiological underpinnings of PTSD and provide a foundation for exploring therapeutic interventions aimed at alleviating the disorder’s impact.

However, the S-R model lacks direct measures of fear responses and may confound results related to depressive-like behavior due to the repeated use of FST as a re-stress cue. The effectiveness of treatments like paroxetine in attenuating both FST- and FS-induced behavioral deficits supports the validity of both models in studying PTSD-related behaviors, although it raises questions about the differential impact on HPA axis dysfunction, as the models may not fully distinguish between the mechanisms of these stress-induced changes [99,100]. Additionally, the initial severe stress sequence and subsequent re-stresses could complicate the interpretation of monoaminergic responses, suggesting that limiting the procedure to a single re-stress might be beneficial [123]. Despite these challenges, the complexity and thoroughness of the stress–re-stress paradigm make it a potent tool in PTSD research, capable of simulating the disorder’s multifaceted nature. Ongoing refinement and incorporation of additional behavioral and physiological measures are essential to enhance its clinical relevance and predictive validity.

The differential contextual odor conditioning (DCOC) paradigm provides a robust framework for exploring the interplay between contextual cues and memory in the context of stress-related disorders, including PTSD [124,125]. Developed by Cohen et al. (2009), the DCOC model exposes rodents to a specific odor, such as cinnamon, across three contexts: aversive (paired with an unpleasant stimulus, e.g., footshock or predator scent), safe (paired with a positive stimulus), and neutral (odor presented without significant associations). Behavioral responses, such as freezing, exploration, and avoidance, are analyzed during re-exposure to assess the impact of these contextual associations.

Traumatic stressors, such as Predator Scent Stress (PSS, see below) and UWT, disrupt the animals’ ability to differentiate between safe and aversive contexts, both when stress precedes training and when introduced after successful training. This disruption reflects the loss of contextual modulation—a phenomenon characteristic of PTSD pathology. While other associative paradigms using stimuli like light or sound provide similar insights, the DCOC model uniquely highlights the role of olfactory cues in triggering trauma-related memories and behaviors.

Beyond behavioral assessments, the paradigm integrates neurobiological analyses, including corticosterone measurements and studies of dendritic remodeling in key brain regions (e.g., prefrontal cortex and amygdala). These assessments provide critical links between observed behaviors and underlying neurobiological mechanisms, enhancing the paradigm’s relevance for understanding PTSD. Despite its limitations, the DCOC model remains an invaluable tool for studying how specific contextual cues modulate memory and emotional responses following trauma.

### 3.4. Time-Dependent Sensitization (TDS)

In the TDS model, animals undergo an initial acute trauma exposure through a sequence of stressors similar to the SPS model, including somatosensory stress (restraint), psychological stress (forced swimming with brief submersion), and exposure to ether vapors [123,124,125,126,127,128]. Following this, the animals are re-exposed to a situational reminder, such as a cue linked to the initial trauma, at later time points (typically 7 and 14 days after the initial exposure). This approach focuses on the memory of the trauma rather than the re-experience of the trauma itself, enhancing its relevance for modeling human PTSD, where the memory of trauma often plays a central role in symptom persistence. The recent research by Viellard et al. (2024) suggests that incorporating elements that allow for dynamic threat detection and avoidance, as seen in naturalistic environments, could enhance the ethological validity of models like TDS, thereby better simulating the complex, real-world conditions that contribute to PTSD [23].

The etiological validity of the TDS model is strengthened by its focus on situational reminders, which are critical components of PTSD in humans. This paradigm simulates the anticipatory anxiety and heightened arousal seen in individuals re-experiencing trauma through reminders, rather than through the reapplication of severe stressors. The delayed and repeated presentation of situational cues allows researchers to model the chronic progression of PTSD, where symptoms often re-emerge or intensify with exposure to trauma reminders. By emphasizing the reactivation of traumatic memory rather than new trauma induction, the TDS model effectively captures the long-term and relapsing nature of PTSD symptoms, thus enhancing its etiological validity.

Studies utilizing the TDS model have consistently found that corticosterone concentrations are elevated immediately after the initial triple stressor phase, while exposure to a situational reminder (RS) results in profound hypocortisolism. This suggests a disturbance in the regulation of the HPA axis, manifesting as enhanced negative feedback upon the re-introduction of the stressful situation. Changes in monoamine concentrations have also been observed, indicating that the TDS model induces monoamine dysregulation. These findings align well with observed neuroendocrine abnormalities in PTSD patients, such as dysregulated HPA axis activity and altered serotonin and dopamine levels, supporting the construct validity of the model. The use of reminders rather than repeated severe stress helps in better understanding the mechanisms of neuroendocrine sensitization and the stress reactivity characteristic of PTSD.

Behavioral analyses in the TDS model typically show that stress-related anxiety is not sustained after the triple stressor phase but becomes most pronounced 7 days after exposure to the RS. This reflects a delayed response to stress, akin to the latent onset of anxiety and hyperarousal seen in PTSD patients. The TDS model also captures aspects of contextual fear, where the situational reminder elicits a marked stress response, mirroring the human experience of intrusive memories and situational triggers. The delayed peak in anxiety-related behavior emphasizes the importance of situational reminders in maintaining PTSD symptoms, which is a core feature of the disorder. The TDS model’s ability to produce sustained stress responses over time without the need for repeated severe stressors offers a valuable perspective on how reminders, rather than re-exposure to traumatic events, can perpetuate PTSD symptomatology.

This integration within the TDS model offers a broader understanding of PTSD, capturing both the initial impact of trauma and the effects of subsequent stress exposures. However, further refinement, such as standardizing situational cues and incorporating dynamic threat detection, will enhance the ethological validity and reproducibility of the TDS model, making it a more robust tool for PTSD research.

### 3.5. Comparative Analysis of PTSD Animal Models: SPS, S-R, and TDS

The SPS, S-R, and TDS models are highlighted together due to their methodological approaches that integrate multiple stressors to simulate PTSD. Despite belonging to different categories—SPS and TDS in Intermediate Complexity Models and S-R in multi-stressor models—their similarities in exploring trauma’s impact on the brain and behavior warrant a comparative discussion. These models are chosen for comparison here (see Table 2) because they collectively offer insights into both the immediate effects of acute stress and the long-term consequences of repeated or situational reminders of trauma.

The SPS model is primarily focused on the acute and persistent effects of a single, prolonged traumatic event, providing valuable insights into extinction retention deficits and altered neurobiological mechanisms within the hippocampus and prefrontal cortex [74,75,76]. On the other hand, the S-R model emphasizes the chronic aspects of PTSD by incorporating repeated stress exposures. It simulates the trigger–response mechanism characteristic of PTSD, primarily inducing anxiety-like states and demonstrating hypocortisolism, though it may not fully capture core PTSD symptoms such as hyperarousal and intrusive memories [82,83,121,122]. Expanding on the foundations laid by the SPS and S-R models, the TDS model integrates aspects from both, focusing on the role of situational reminders in the chronic progression of PTSD. The TDS model incorporates intense acute stressors followed by situational reminders, leading to a disruption in HPA axis regulation and sustained PTSD symptoms [123,126,128].

To summarize this section, it might be speculated that the reproduction of a complex response to stress in humans and the predisposition to the development of PTSD within a certain model may be achieved by combining different stressors. It does not always (and rather often does not) reflect natural stressors in humans, but it has been shown that the combination of stressors of different natures may result in behavioral and cognitive typical for specific human categories. For example, a recent study showed that the military-relevant acute stress response can be induced by imposing rats to the three-stressor treatment, namely, inescapable shock plus predator exposure plus UWT [129].

## 4. Social Interaction Models

While the preceding sections have categorized PTSD models based on the complexity and number of stressors involved, it is essential to recognize that social stress models warrant a distinct discussion due to the unique role social factors play in the development of PTSD. Social stressors, such as isolation, social defeat, and housing instability, influence PTSD through mechanisms that are fundamentally different from those involved in purely physical or psychological stressors. These models are pivotal in understanding the interplay between social environments and PTSD, offering insights into how social contexts can either mitigate or exacerbate stress responses. By addressing social stress models as a separate category, we aim to emphasize the significance of social factors in PTSD research and to highlight the need for tailored interventions that address the social dimensions of trauma.

### 4.1. The Role of Social Interaction

The role of social interaction in the development of physical, cognitive, and emotional capacities is of paramount importance, particularly in young mammals, including rodents. Social play behavior is not simply a pleasurable activity; it is a highly vigorous and rewarding social behavior that is essential for the proper development of brain and behavior [130]. It is especially prevalent during juvenile and early adolescent stages, and its disruption can lead to deficits in social, cognitive, and emotional processes, which are often observed in various pediatric psychiatric disorders [130].

The rewarding aspects of social play are modulated by key neurotransmitter systems such as opioids, endocannabinoids, dopamine, and noradrenaline. The nucleus accumbens has been identified as a crucial site for opioid and dopamine modulation of social play behavior, underpinning the rewarding and motivational elements of social play [130]. Furthermore, endocannabinoids are believed to exert their effects primarily via the basolateral amygdala, which also plays a key role in the noradrenergic regulation of social play. Viviana Trezza and colleagues (2010) highlighted that social play behavior, akin to natural and drug rewards, activates the brain’s reward systems, particularly those involving opioids and endocannabinoids, making social play a crucial component in building behavioral flexibility and acquiring social competence [131]. Such capabilities are vital for maintaining group cohesion and resilience against stress, providing a framework for understanding how neurochemical pathways, also implicated in drug reward mechanisms, underlie social play. This connection underscores the critical nature of social play in promoting emotional and cognitive health, which can be compromised in conditions like PTSD when social engagement is disrupted [131]. Collectively, social play is the result of coordinated activity across a network of corticolimbic structures, which are heavily modulated by monoaminergic, opioid, and endocannabinoid systems, contributing to behavioral flexibility, social competence, and stress resilience.

Recent research has further emphasized that rodents are not only highly cooperative but are also motivated to perform prosocial actions in response to the distress of conspecifics, such as opening a door to release a trapped peer [132]. Such prosocial tendencies indicate the intrinsic value and reward associated with social interaction. Unrestricted social play, in particular, has been shown to play a crucial role in the development of inhibitory synapses in the PFC, thus contributing to enhanced cognitive abilities in adulthood [133]. Specific synaptic alterations in the PFC, linked to unrestricted social play, can yield complex behavioral outcomes, further illustrating the importance of early social experiences for healthy neural development.

### 4.2. Social Stressors in Animal Research

Social stressors, such as social isolation (SI), housing instability (HI), and juvenile social exploration (JSE), are crucial for modeling aspects of mood disorders and PTSD. These stressors highlight the underlying mechanisms of these conditions, showcasing the intricate effects of environmental influences on susceptibility to mood disorders and PTSD. Exploring behavioral, physiological, and neurobiological responses to these stressors deepens our understanding of these complex disorders and aids in developing more effective interventions [134].

Rodents exposed to SI demonstrate behavioral deficits similar to PTSD symptoms, including enhanced fear responses and compromised fear extinction memory [134]. This observation offers insights into vulnerability to PTSD, reflecting the impact of sustained stress. Socially isolated mice also undergo neurobiological alterations, such as reductions in corticolimbic allopregnanolone levels and changes in fear-related neurocircuitry [134].

The HI model in rats and the SI model in mice both generate stress-related outcomes such as elevated corticosterone levels, anxiety, and freezing behavior, aligning with PTSD-like symptoms [135,136]. In social isolation models, simultaneous changes were observed not only in corticosterone but also in other hormones like adrenocorticotrophic hormone, leptin, and growth hormone [137]. Similarly, early-life stress (ELS), particularly maternal separation, serves as a model for childhood trauma, affecting susceptibility to PTSD in later life by inducing long-term behavioral, hormonal, and brain structural and functional changes [134]. Social defeat stress (SDS), which includes variations such as the resident-intruder, witnessed social defeat, and cage-within-cage resident–intruder models [138], effectively models PTSD-like symptoms by highlighting social avoidance and other associated behavioral outcomes, including anxiety, heightened fear, and exaggerated startle responses. In SDS paradigms, an experimental rodent (the intruder) is placed into the home cage of a larger, aggressive resident rodent, leading to a defeat episode characterized by physical subordination and social stress. This exposure is repeated over multiple days, inducing chronic stress conditions that mimic PTSD-like symptoms. Between confrontations, the intruder is housed in close proximity to the aggressor but separated by a perforated barrier, providing continuous sensory exposure without physical interaction. This process effectively mimics chronic exposure to psychosocial stress, inducing enduring behavioral and physiological changes that are highly relevant to PTSD [139].

SDS models are comprehensive in their ability to assess PTSD-related markers, including behavioral outcomes such as social avoidance and physiological effects such as increased corticosterone and altered blood and brain biomarkers [140,141]. Standardized SDS protocols have been shown to induce stable and reproducible stress responses, allowing researchers to differentiate between individuals who are susceptible to or resilient against PTSD-like conditions. Such standardization is critical for evaluating both behavioral and physiological outcomes, providing insight into the full spectrum of PTSD [142,143].

The JSE test, introduced by Dr. Sandra File and Dr. J.R.G. Hyde in 1978, revolutionized anxiety-like behavior assessment by utilizing social interaction as a measure [144,145,146]. Central to JSE is the introduction of a novel juvenile rat to adult rats in a neutral setting, assessing social engagement as an indicator of anxiety levels. JSE, particularly when paired with an immobility session, enriches our understanding of various phenomena relevant to PTSD, including learned helplessness, stress-induced analgesia, and resilience mechanisms (reviewed in [3]). In this setup, both control and experimental rats encounter novel juveniles in a clean cage without food or water. Subjects acclimate for 60 min before interacting with the juvenile, with procedures repeated to gauge baseline and post-IS social behaviors [147,148]. JSE has provided significant insights into social avoidance and stress-related neuroinflammation but faces validation challenges in prolonged stress incubation models and conspecific exploration (reviewed in [3]).

However, SI models have several limitations that may impact the validity and applicability of research findings (Table 3). A key issue is their ethological relevance. SI in rodents often yields results that are difficult to generalize to human PTSD due to differences in social structures and responses to isolation between species. While both humans and rodents are inherently social, prolonged isolation in rats can lead to behaviors, such as increased aggression or anxiety, that may not accurately represent the social withdrawal or loneliness experienced by humans with PTSD. In these models, the observed behaviors may result more from the stress of isolation itself than from PTSD-like symptoms.

The behaviors exhibited by socially isolated rats, such as increased aggression or anxiety, may not stem directly from PTSD-like symptoms but from the stress of isolation itself. This can make it difficult to discern whether the behaviors result from the model’s intended stressor (simulating PTSD) or simply a reaction to the unnatural conditions of isolation. Moreover, SI can induce a broad spectrum of stress-related behaviors and (patho)physiological changes, many of which overlap with symptoms of other disorders such as depression and anxiety. This lack of specificity can make it challenging to use SI models to study PTSD exclusively, as the induced symptoms may not uniquely align with those of PTSD.

Generalizability issues also pose a challenge. The effects observed in SI rats may vary depending on the age at which isolation begins, the duration of isolation, and the individual animal’s genetic background and prior social experiences. These variables can lead to inconsistent results across studies, complicating the generalizability of findings. For this reason, standardized social housing conditions are particularly important both preceding and during the experiment. To avoid confounding influences of social isolation or the formation of social hierarchies, it is recommended to house males with sterilized females [149]. Control group issues further complicate the validity of findings. In many studies, the comparison between SI rats and those housed in groups might not adequately control for variables other than social interaction, such as environmental enrichment and space per animal. This can lead to confounding results where differences could be attributed not only to SI but also to other uncontrolled factors.

Although most social defeat stress (SDS) models violate the criteria of a single traumatic exposure, SDS may be relevant for socially induced or combat-related PTSD, which typically involves multiple exposures. SDS may also be particularly relevant for comorbidity with depression. An important limitation of the SDS model is the lack of protocol for social stress in females [22]. This broad spectrum of social stressor models, from social isolation to JSE, underscores the complex interplay between social environment and the development of mood disorders and PTSD, highlighting the need for comprehensive approaches in research and intervention strategies.

## 5. Predator-Based Models

**Figure 4:**
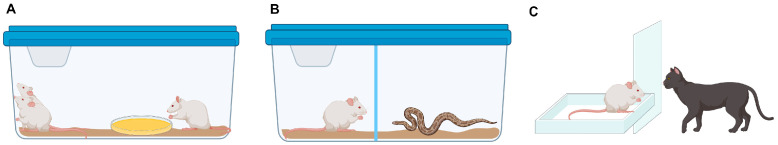
A simplified representation of predator-based models. (**A**) The animal is exposed to a predator scent or odor (urea, fur, or collar) but is not exposed to direct contact with the predator. This method provides the highest level of protection for the animal. (**B**) The animal is exposed to the direct view of the predator (and often in the common space allowing transmission of scents and dangerous sounds), but direct contact between the animal and the predator is blocked, thus providing an intermediate danger level. (**C**) The animal is exposed to direct contact with the predator. In this method, the only barrier between the animal and the predator is a transparent shield, which partially blocks the contact but does not prevent attack. The methods simulate the most dangerous situations.

Predator stress paradigms involve exposing animals to stressors that are either direct (unprotected exposure to a predator), indirect (exposure with a physical barrier), or olfactory (exposure to predator scent). These stressors are inescapable and unpredictable, providing strong ethological validity [22,74,125,136,150,151,152,153,154,155,156,157,158,159,160,161,162,163,164,165,166,167,168,169,170,171,172,173,174,175,176,177,178,179,180,181,182,183,184,185,186,187,188,189,190,191,192,193,194,195,196,197,198,199,200,201,202]. Figure 4 schematically represents the most often used variants of the model.

### 5.1. The Predator Scent Stress (PSS) Model

The Predator Scent Stress (PSS) model is a well-established approach for inducing acute stress responses that simulate life-threatening situations. This model primarily involves the exposure of rodents to predator odors, such as cat or fox urine, which are known to trigger (patho)physiological and behavioral responses that resemble the manifestations of PTSD in humans.

When rodents are exposed to predator odors, such as cat or fox urine, they exhibit a range of stress responses, including enhanced negative feedback in the HPA axis, increased amygdala activity, and altered neurotransmitter levels in the hippocampus and prefrontal cortex [150]. These responses mimic the neuroendocrine and neurobiological changes seen in humans under acute stress. Additionally, predator stress can induce long-term brain inflammation, which has been shown to be responsive to anti-inflammatory treatments [150]. These physiological effects are associated with behavioral changes, such as hyperarousal, avoidance behaviors, exaggerated fear responses, and impaired fear extinction—all of which are key features of PTSD [125,136,151].

PSS models utilize both natural and synthetic stimuli to simulate life-threatening scenarios and study stress responses. Natural cues, such as predator urine, fur, collars, and bedding, are highly effective in eliciting innate fear responses, including freezing, reduced motor activity, and hypervigilance. These cues are ecologically valid, reflecting real-life predator threats in the rodents’ ancestral environments. For example, exposure to cat urine consistently induces defensive behaviors like reduced grooming and reproductive activity, as well as persistent hyperarousal [174,175,176,177,178,179,180]. In contrast, synthetic compounds, such as 2,4,5-trimethylthiazoline (TMT), offer greater experimental control over intensity and duration but do not fully replicate the behavioral profile seen with natural stimuli. While TMT induces freezing behavior and alters stress-related gene expression, it is often perceived as an unpleasant odor rather than a genuine predator threat. Studies comparing cat urine and TMT show that natural cues uniquely activate hypothalamic regions critical for defensive behaviors, whereas TMT falls short in triggering avoidance and risk assessment behaviors [174,175,176,177,178,179,180]. Similarly, compounds like 2-phenylethylamine cannot replicate the complete defensive response profile seen with predator urine, highlighting limitations in synthetic analogs [181].

PSS models have demonstrated significant utility in modeling PTSD-like symptoms by utilizing natural and synthetic olfactory stimuli that simulate life-threatening situations. They are also characterized by their ability to identify resilient and non-resilient subgroups, providing insight into the heterogeneity of PTSD responses within populations. However, there are also limitations, such as individual variability, sex-specific responses, and challenges in ensuring consistency and robustness of PTSD-like outcomes across different studies.

The ethological validity of predator-based models is rooted in their use of naturalistic predator threats, such as urine, fur, collars, and bedding, or even direct exposure to live predators. These stimuli are effective in replicating real-life threat scenarios that rodents might face in their natural habitats, thereby making the models ecologically relevant. Such naturalistic exposures—whether direct confrontation or olfactory exposure—elicit innate defensive behaviors, such as freezing, avoidance, reduced grooming, and heightened vigilance, which mirror the responses that rodents would exhibit when faced with real predator threats. By integrating both natural and controlled synthetic cues (e.g., TMT), predator-based models achieve a balance between ecological relevance and experimental control, making them particularly valuable in the study of PTSD-like symptoms in a laboratory setting. For instance, snake odor elicits anxiety-like behaviors (e.g., changes in sniffing, grooming, and digging frequency) in CD-1 and DBA2 mice, though it does not significantly affect analgesia [170]. In Swiss mice, snake odor failed to induce defensive behavior or changes in Fos expression within the hypothalamic circuit, unlike exposure to cat odors [171]. Conversely, *Acomys cahirinus* mice showed both behavioral and physiological fear responses to snake odors, but not to owl calls, which suggests that the ancestral predatory environment influences these responses [172].

The etiological validity of the PSS model is rooted in its ability to reproduce the uncontrollable and life-threatening nature of predation, similar to traumatic experiences faced by humans. These threat scenarios can mirror human PTSD exposure by replicating unpredictable and inescapable stressors. The diverse predator cues—from direct predator exposure to olfactory stimuli—mimic the diverse triggers that can lead to PTSD, such as unpredictable threats and overwhelming fear [150]. The use of different forms of predator exposure (urine, fur, collars, or auditory sounds like owl calls) provides a rich context for studying the etiology of PTSD-like symptoms.

Predator-based models consistently elicit behavioral changes associated with PTSD-like symptoms, including hyperarousal, avoidance behaviors, exaggerated fear responses, and impaired fear extinction. Exposure to predator scents, such as cat urine, significantly reduces general motor activity, grooming, and reproductive behaviors while provoking sustained defensive reactions, even in rodents without previous predator encounters [164,165]. Chronic exposure to predator odors, such as repeated exposures to predator urine over weeks, has been shown to induce substantial anxiety/depressive symptoms and reduced blood glucocorticoid levels [163,168,169]. Synthetic analogs like TMT can also elicit fear-like responses, including freezing behavior and differential gene expression of stress-related genes in rodents [173,174]. However, TMT is often argued to act more like an unpleasant odor than a true fear-inducing stimulus, with cat odors eliciting more profound defensive and neural responses [173,187].

The construct validity of predator-based models is demonstrated through their ability to replicate specific molecular and neurobiological changes observed in PTSD. These include enhanced negative feedback in the HPA axis, altered neurotransmitter levels, and increased amygdala activity, mirroring key neuroendocrine pathways in PTSD patients [150]. Furthermore, predator stress models differentiate between resilient and non-resilient phenotypes based on distinct molecular patterns. For example, animals exhibiting PTSD-like symptoms display elevated expression of allograft inflammatory factor 1, downregulation of CX3C chemokine receptor 1, and changes in microglial morphology, which are absent in resilient animals [74]. Such molecular changes underscore the utility of predator-based models in exploring stress-induced pathology and therapeutic strategies. In mostly stress-sensitive animals, there was a marked downregulation of neuropeptide Y in the hippocampus, periaqueductal gray, and amygdala, while such changes were absent in resilient groups [152]. Additionally, elevated leptin levels and an increased testosterone/corticosterone ratio were found to distinguish stress-resistant rats from their susceptible counterparts [154]. Pre-stress neuroinflammation also emerged as an important factor for identifying stress resilience or susceptibility—especially in the prefrontal cortex [155]. However, sex-specific differences were noted but not fully explained by sex alone, as molecular changes were consistent across animals of the same sex [155].

While the PSS model has notable strengths, there are challenges to its implementation. Sex-specific responses complicate the interpretation of these models, with less frequent extreme behavioral responses in females compared to males, highlighting the need for more consistent model applications and thorough phenotyping criteria [198,199,200,201,202].

In summary, PSS models provide a highly ethologically valid approach to modeling PTSD-like symptoms by using natural predator cues to replicate life-threatening situations. Their etiological validity lies in their effective replication of unpredictable, inescapable trauma akin to human experiences of PTSD. The behavioral outcomes observed are consistent with PTSD-like responses, including hyperarousal, avoidance behaviors, and fear responses. The model’s construct validity is demonstrated through its induction of neurobiological changes in stress pathways, such as those involving neuropeptide Y, leptin, and HPA axis regulation. Nevertheless, individual variability and sex-specific responses present challenges to the consistent application of this model, underscoring the need for further standardization and refinements in study design and animal selection.

### 5.2. The Direct Confrontation with Predators

The direct confrontation of rodents with natural predators offers several unique advantages as a model of traumatic or stressful experiences. For example, the predator-based model utilizes a 31-day protocol where rats encounter a predator (an adult female cat) on two separate occasions, with a 10-day interval between exposures, combined with chronic social instability through daily changes in cage mates. This complex model aims to simulate PTSD-like symptoms by mimicking re-experiencing symptoms and providing a chronic, mild stressor akin to the fluctuating social environments often linked with PTSD in humans. Rats subjected to this model exhibit a range of (patho)physiological and behavioral changes, including heightened anxiety, amplified startle responses, cognitive impairments, and increased cardiovascular sensitivity [183,184].

Mice exposed to direct contact with a snake demonstrate anxiety- and panic-like behaviors, such as freezing and spatial avoidance, along with changes in Fos protein levels in limbic structures, including the amygdala, hypothalamus, and periaqueductal gray matter [185,186,187]. However, significant analgesic responses to snake predators were noted in juvenile but not adult voles, with females exhibiting greater sensitivity to non-opioid analgesia compared to males [188].

A particularly intense version of the snake-based model of psychogenic stress involves rats witnessing a littermate being attacked and consumed by a snake, such as the Indian python (*Python molurus*) or reticulated python (*Malayopython reticulatus*) [189,190]. In this model, rats are placed in a terrarium with a hungry python, where they can freely explore the environment but experience severe psychogenic trauma when one of the rats is killed by the snake, simulating a life-threatening event. This form of stress exposure was found to increase anxiety in the open field and elevated plus maze tests. Blocking galanin receptors in the brain aggravated these behavioral changes, suggesting that the endogenous pool of galanin helps prevent excessive CNS responses to stressful stimuli, characteristic of PTSD [191]. Psychogenic trauma also led to increased locomotor activity in the open field test in rats that had received LPS injections during the early postnatal period [187]. These behavioral alterations were associated with changes in the expression of the NMDA receptor subunit GluN2B in the ventral hippocampus—a region primarily involved in emotional and motivated behaviors—while no changes were observed in the dorsal hippocampus, which is more related to cognitive functions, learning, memory, and spatial navigation [192].

Additionally, direct confrontation with predators resulted in both immediate behavioral reactions and long-term metabolic alterations. During the traumatic event, pronounced fear responses were observed, including “freezing”, clustering together, rearing (standing on hind legs), prolonged and altered grooming, and, in some cases, agitated, uncontrolled movement throughout the terrarium. Following the exposure, rats that were repeatedly exposed to snake aggression showed significant metabolic changes, including decreased HDL cholesterol levels and increased serum triglycerides, which persisted for at least six months post-exposure. These metabolic changes are associated with an increased risk of cardiovascular disorders—similar to those observed in humans suffering from PTSD [186].

Other versions of the predator confrontation model involve exposing rats to snakes without direct physical contact, allowing visual, olfactory, and auditory perception of predators, such as ball pythons (*Python regius*), black-footed ferrets, and domestic cats [193]. Behavioral analyses of Sprague Dawley rats in such experiments were conducted using the EPM and ASR tests, with k-means principal components analysis revealing high variability in the behavioral outcomes of stressed animals.

Exposure to live ferrets led to enhanced activation of stress-related brain regions, including the HPA axis, and increased Fos protein expression in areas such as the medial amygdala and dorsomedial periaqueductal gray. This activation was notably higher than standard laboratory stressors like footshock, with ferret exposure inducing approximately twice the level of Fos activation in these regions [194]. Moreover, exposure to a live predator elicits more intense activation of stress-related brain areas compared to exposure to odor alone, triggering a cascade of events in the amygdala that ultimately leads to prolonged sensitization and the emergence of a PTSD-like phenotype [194,195].

Direct confrontation models excel in replicating life-threatening situations akin to human traumatic events. They offer significant etiological and ethological validity by mirroring predation threats naturally encountered by animals. However, the challenges are notable—only a part of rodents develop full PTSD-like phenotypes, while others show intermediate or minimal symptoms, pointing towards genetic variability as a determinant of PTSD vulnerability. Additionally, the use of live predators raises ethical concerns regarding the severity of stress imposed on animals and the inherent variability in predator behavior that might complicate standardization and reproducibility. Nonetheless, these models are valuable tools for understanding extreme stress and stress-induced psychogenic trauma.

### 5.3. Integrating Military-Relevant Trauma into Predator-Based Models

The etiology of PTSD in military contexts involves a multifactorial interplay of combat exposure, pre-military vulnerabilities, military adjustment, social support, and treatment engagement. Insights from combat operations, such as Operation Enduring Freedom (OEF) and Operation Iraqi Freedom (OIF), underscore the unique challenges faced by military personnel and their psychological impacts. These findings provide a critical foundation for developing animal models with strong ethological and etiological validity, replicating the multifaceted stressors associated with combat trauma.

Combat exposure remains the most significant predictor of PTSD. Service members in high-intensity environments encounter a range of traumatic experiences, including witnessing death, handling severely injured individuals, and facing life-threatening situations. For instance, 95% of Army Soldiers deployed in Iraq reported seeing dead bodies compared to 39% in Afghanistan [203]. The elevated exposure to violent and fatal incidents in Iraq aligns with higher reported PTSD rates among its veterans compared to those who served in Afghanistan. Prolonged exposure to forward areas, repeated incidents of rocket or mortar fire, and sustained periods of hypervigilance amplify risk. These cumulative exposures underscore the necessity of animal models capable of replicating both acute and chronic trauma exposure while maintaining experimental control and translational relevance [203].

Pre-military factors, including genetic predispositions, early-life trauma, and prior exposure to adverse events, also significantly influence PTSD susceptibility. These factors interact with combat-related stress, heightening vulnerability. Early-life stress, such as maternal separation, neglect, or physical abuse, alters the stress response system, predisposing individuals to heightened anxiety and maladaptive responses to later trauma [203]. Although such factors may not directly cause PTSD, their interaction with high-stress demands in military settings can exacerbate outcomes. Sociodemographic factors further compound risk, with female service members and those of Hispanic and Asian Pacific ethnicity showing specific susceptibility to PTSD [204,205]. Variables such as lower levels of education and being unmarried may also increase risk, as they often correlate with reduced access to coping resources and social support [203].

Military adjustment plays a pivotal role in resilience to PTSD. Factors like unit morale, strong leadership, and cohesive social networks within military units serve as protective factors against the effects of trauma [206]. Conversely, low morale, poor unit cohesion, and family-related stressors during service increase susceptibility to PTSD which is further exacerbated by post-military stress, including financial instability and difficulty reintegrating into civilian life [203,204].

Social support remains one of the most critical protective factors against PTSD. Strong camaraderie and morale within units reduce the likelihood of PTSD onset, while the absence of post-trauma social networks significantly exacerbates symptoms. Testing animal models in group settings rather than isolation provides an opportunity to explore the role of social factors, as both rats and humans are inherently social beings. These experiments could replicate the dynamics of unit cohesion and isolation in military PTSD, shedding light on the therapeutic value of post-trauma social reintegration [206,207,208].

Delayed PTSD onset, often observed in OEF/OIF veterans, further complicates the disorder’s trajectory. In many veterans, delayed-onset disorders are often qualified as non-PTSD but so-called sub-syndromal PTSD [209]. Symptoms can emerge months after deployment, likely as initial coping mechanisms become overwhelmed or as life changes resurface combat memories. Comorbid conditions, such as depression, substance abuse, and interpersonal conflicts, are also prevalent, complicating diagnosis and treatment. Animal models incorporating long-term behavioral and physiological assessments can help address this complexity, providing insights into delayed-onset PTSD and its contributing factors.

The progression from predator-based stress models to more complex combat-relevant paradigms reflects the continuous refinement of preclinical approaches to replicate military trauma with high ethological validity. Initial studies by Genovese et al. demonstrated that behavioral disturbances occur after a single predator exposure or repeated exposures to three predator species. While these models effectively induced fear-based behaviors, they lacked elements of military-specific trauma, such as prolonged stress and psychological unpredictability [210,211].

Building on predator exposure, a two-stressor model was developed by combining predator encounters with UWT. UWT introduced ecological relevance by simulating perceived threats to life, physiological distress, and unpredictability, thereby reflecting life-threatening military scenarios [211]. Further refinement led to a three-stressor model, incorporating inescapable footshock alongside predator exposure and UWT. This non-injurious but physically painful stressor heightened the unpredictability and complexity of the environment. Henschen et al. (2023) demonstrated that this model produced the greatest behavioral disturbances, both in the number of affected variables and the magnitude of stress effects [129]. Moreover, the duration of predator exposure influenced behavioral outcomes, with shorter exposures eliciting distinct deficits compared to prolonged encounters [129,136,212]. These multi-stressor models demonstrate strong ethological validity by replicating post-trauma phenotypes, including fear responses, cognitive impairments, and physiological stress markers.

The “Vital Stress” model proposed by Tsikunov and colleagues provides an alternative by refining predator exposure without adding additional stressors [189,190,191]. In this paradigm, pairs of female rats are exposed to a tiger python in a terrarium, where one rat becomes prey while the survivor observes from behind a transparent barrier for 30–40 min [189,190]. This setup combines the stressors of life-threatening danger and witnessing conspecific loss, eliciting pronounced fear behaviors such as freezing, huddling, altered grooming, and frantic movement. By focusing on modifying the predator exposure mechanism, this model enhances the realism of the threat and provides a focused simulation of trauma that closely aligns with military PTSD scenarios.

The current progression from simple predator-based stress models to multi-stressor paradigms has advanced our ability to replicate combat-related PTSD in laboratory settings. To enhance these models, researchers should incorporate additional elements, such as social interactions and early-life stress. Testing animals in groups rather than individually can simulate both the protective and exacerbating effects of social dynamics. Social bonds within groups may serve as a buffer against trauma, while witnessing conspecific deaths may worsen outcomes, reflecting the dual role of social factors in PTSD [213]. Incorporating early-life stress paradigms into a subset of animals can replicate pre-military-like vulnerabilities. Rodents exposed to maternal separation, neglect, or unpredictable stress during early development display altered stress responses and heightened anxiety, paralleling human predispositions to PTSD [203]. Overall, by enriching these models with social and developmental components, researchers can better replicate the multifactorial etiology of PTSD.

### 5.4. The Common Features of Predator-Based Models

Predator-based models demonstrate high ethological validity as they use natural threats such as predator urine, fur, or bedding, which closely replicate the unpredictable and inescapable nature of real-life predatory threats. The behaviors elicited, such as hyperarousal, avoidance, and freezing, are innate responses observed in rodents in the presence of predators. Versions of the model using live predator encounters (e.g., cat or snake exposure) provide a naturalistic setting that mirrors the predation threats animals face in the wild, further strengthening the ecological relevance of these models.

Predator stress paradigms exhibit etiological validity by reproducing inescapable, life-threatening conditions that are akin to human experiences of extreme fear and perceived danger—core components of trauma exposure in PTSD. The uncontrollable nature of predator stress, particularly in direct or olfactory exposures, mimics the unpredictability and intensity of human traumatic events, thus helping to model the onset of PTSD-like symptoms [150].

Behavioral outcomes in predator stress models include hyperarousal, exaggerated fear responses, avoidance behaviors, impaired fear extinction, and reduced general activity, all of which are consistent with PTSD symptomatology. Exposure to predator scent, like cat urine, leads to decreased grooming and reproductive behaviors and sustained defensive reactions, even in rats without previous predator exposure [164,165]. Chronic exposure also results in anxiety/depressive symptoms and altered motor activity in rodents, paralleling persistent anxiety and arousal in PTSD [163,168,169]. Predator-exposed rats also show enhanced startle responses and impaired performance in fear extinction tasks, behaviors commonly linked to PTSD [183,184].

Construct validity is demonstrated as predator stress paradigms replicate key neurobiological changes observed in PTSD, including HPA axis dysregulation, amygdala hyperactivity, altered neurotransmitter levels, and markers distinguishing resilience from susceptibility [150,151,152,153,154,155,169,214]. Thus, the predator-based stress paradigms effectively illustrate the individual variability in stress responses by distinguishing between resilient and non-resilient phenotypes. This differentiation reflects the heterogeneity in PTSD phenotypes found in human populations. Neurobiological and behavioral markers have been identified to differentiate these phenotypes. Non-resilient animals—those developing PTSD-like symptoms—display an elevated expression of allograft inflammatory factor 1 and a downregulation of CX3C chemokine receptor 1 in the hippocampus, alongside microglial morphological changes [74]. These markers are not observed in resilient animals. Additionally, non-resilient animals show significant downregulation of neuropeptide Y in the hippocampus, periaqueductal gray, and amygdala, while resilient subgroups do not exhibit such changes [151,154]. Another distinguishing factor is the hormonal profile; stress-resistant animals are characterized by higher levels of leptin and an increased testosterone/corticosterone ratio, whereas stress-susceptible animals exhibit lower levels of these markers [154]. Pre-stress neuroinflammation—particularly in the prefrontal cortex—has also been found to play a crucial role in determining whether an animal is resilient or susceptible to stress, while systemic (peripheral) inflammation markers do not differ significantly between phenotypes [155]. Moreover, Komelkova et al. (2020), in their study using the PSS model, noted significant metabolic differences between resilient and non-resilient animals. Using the hexobarbital sleep test to predict PTSD susceptibility in rats, they found that slow metabolizers were more vulnerable to developing PTSD-like symptoms, as indicated by higher anxiety levels and increased corticosterone levels [214]. Behaviorally, resilient animals maintain better performance in fear extinction tasks and exhibit less pronounced hyperarousal and avoidance behaviors compared to their non-resilient counterparts. The ability of predator-based models to distinguish between these phenotypes makes them valuable for exploring the underlying neurobiological mechanisms and the diverse trajectories of PTSD, as well as for evaluating treatments that might enhance resilience or mitigate susceptibility to trauma-induced conditions.

It is important to emphasize that not all rats exposed to predator stress develop PTSD-like symptoms, which is actually reflective of the human population, where only a small percentage of individuals develop PTSD following trauma exposure. It has been shown that only 25% of rats display PTSD-like symptoms, whereas many show minimal (25%) or intermediate (50%) responses. This variability is not a limitation but rather an accurate representation of the diversity of responses observed in human populations and underscores the genetic complexity of PTSD susceptibility [156,183,184,196]. Additionally, Cohen and colleagues established “cut-off behavioral criteria” to classify animals as susceptible or resilient based on extreme reductions in exploratory behavior and increased arousal 7–90 days post-stress [197]. This approach addresses the individual variability in responses [22].

Overall, predator stress models are valued for their etiological relevance, producing robust behavioral and biological phenotypes that are sensitive to treatments like chronic SSRIs and allowing for the identification of animals susceptible to PTSD-like conditions versus those that are resilient [22]. However, challenges remain, such as the presence of sex-specificity in the behavioral response to the predator scent stressor, with less frequent extreme behavioral responses in females compared to males [198,199,200,201,202]. The variability in predator stress protocols also complicates findings, sometimes necessitating the integration of secondary stressors to enhance model efficacy [183]. Differences in rodent species and strains hinder reproducibility across laboratories [196,199]. Moreover, predator stress models do not fully account for components of the clinical syndrome involving episodic (autobiographical) memory alterations, such as memory impairment for events immediately before and after the trauma and dissociation of the traumatic memory from ordinary autobiographical memory (reviewed in [201]).

Finally, while predator stress models provide valuable insights into innate fear responses in animals, their applicability to human PTSD may be limited. Human innate fears, such as those triggered by predators, pain, heights, rapidly approaching objects, and ancestral threats like snakes and spiders [202], are often managed or mitigated in modern contexts, particularly through medical interventions like anesthetics for pain. Given that fear of pain is likely one of the most prevalent, but is frequently controlled in clinical settings, the relevance of predator-related fear in these models may not fully capture the most common or impactful fears experienced by humans. Therefore, predator stress models may not effectively replicate or simulate the predominant natural fears in humans, suggesting a need for caution in their use as analogs for human PTSD. Table 4 summarizes the pros, cons, and limitations of the predator-based models used in PTSD studies.

## 6. Pharmacological and Genetic Approaches in PTSD Research

Pharmacological and genetic models are primarily utilized as tools in PTSD research to investigate the role of specific pathways in the pathogenesis of the disorder and to assess the efficacy of various medications [215]. These models are invaluable for examining how alterations in neurochemical systems and genetic predispositions contribute to PTSD, taking into account that the contributing roles of different neuroanatomical structures like the amygdala, hippocampus, hypothalamus, and others to the onset of the pathological state are largely elucidated [216]. By targeting specific pathways, researchers can dissect the contributions of individual neurobiological mechanisms to the overall disorder. Additionally, pharmacological models enable the evaluation of therapeutic interventions, providing insights into their potential effectiveness and mechanisms of action. However, both pharmacological and genetic models often focus on single characteristics of PTSD, rather than replicating the full complexity of the disorder, thus serving as complementary approaches within a broader research framework.

### 6.1. Pharmacological Approaches and Validation

Pharmacological models of PTSD involve using specific agents to impact pathophysiological mechanisms associated with the disorder. While no single pharmacological model fully replicates PTSD, these approaches manipulate various neurochemical systems to induce conditions or symptoms reflective of PTSD. Such models provide insights into the disorder’s underlying mechanisms and potential treatments.

Pharmacological models rely less on ethological validity, as they focus on specific neurochemical manipulations rather than naturalistic stimuli or behaviors. However, the behaviors observed in response to the pharmacological interventions align with PTSD symptoms, such as hyperarousal, anxiety, and avoidance (Table 5).

These models simulate PTSD-like symptoms by directly altering neurochemical systems implicated in human PTSD. For example, metyrapone disrupts glucocorticoid synthesis by inhibiting 11β-hydroxylase, simulating the HPA axis dysregulation often seen in PTSD. This approach elucidates how reduced glucocorticoid levels influence anxiety and cognitive functions [97,217,218,219]. Similarly, corticosterone administration models prolong glucocorticoid exposure, reflecting chronic stress conditions that can induce PTSD-like symptoms [155,220,221]. CRF injections mimic an elevated stress response, inducing anxiety-like behaviors and disrupting the HPA axis [222,223,224]. These interventions replicate the biochemical pathways involved in PTSD, offering etiological relevance.

The pharmacological approaches produce a range of behaviors that model PTSD symptoms, including hyperarousal and anxiety induced by anxiogenic agents like FG-7142, a GABA_A_ receptor inverse agonist, and yohimbine, an alpha-2 adrenergic receptor antagonist. These substances reflect GABAergic and adrenergic contributions to PTSD [225,226,227,228,229]. Mood, perception, and social behavior alterations are observed with MDMA, which has neurotoxic effects at high doses [226]. Pimavanserin, a selective 5-HT2A inverse agonist, reverses persistent stress effects, demonstrating the serotonin system’s involvement in PTSD [230]. These outcomes provide behavioral parallels to PTSD symptoms observed in humans, aiding the study of underlying mechanisms and potential interventions.

Pharmacological models demonstrate construct validity by inducing molecular and cellular changes reflective of PTSD mechanisms. Regional expression differences in GABA_A_ and NMDA receptors in the ventral hippocampus, along with altered electrical activity in the dorsal dentate gyrus, influence pharmacological treatment efficacy [57]. Inter-individual variation in Ca^2+^/calmodulin-dependent protein kinase II (CaMKII) expression in the hippocampus highlights differences in stress susceptibility and resilience [232]. These findings emphasize the role of hippocampal excitation–inhibition balance in regulating pharmacological responses, supporting the need for individual behavioral profiling to improve translational relevance.

Despite their utility, pharmacological models face significant challenges [231,232,233,234,235]. Variability in treatment response is a major issue, as current medications, such as SSRIs, are effective in only about 50% of patients. Regional hippocampal signaling differences distinguish responding versus non-responding animals, complicating validation efforts [56,231]. The complex neurobiology of PTSD means a single molecular deficit is unlikely to serve as a universal biomarker, as shown by meta-analyses of symptom-specific treatment effects [235]. Furthermore, the stagnation in PTSD pharmacotherapy development is evident, with few innovations over the past 40 years. Medications like sertraline and paroxetine have shown limited effectiveness, and major pharmaceutical companies have reduced investment in psychiatric drug development [233,234]. Notably, promising treatments like ketamine and MDMA-assisted psychotherapy did not originate from basic research, highlighting the complexities of developing effective pharmacological therapies for PTSD.

The pursuit of precision medicine in PTSD research is crucial. Advances in animal models, biosignature identification, and innovative approaches are needed to address the complex neurobiology of PTSD effectively [134].

Pharmacological validation remains essential for assessing animal models of PTSD but is fraught with challenges due to the complexity of the disorder and interspecies differences. While pharmacological models provide valuable insights into neurochemical pathways, achieving construct validity is difficult given the incomplete understanding of PTSD’s neural basis. The pursuit of more precise models and individualized approaches is critical for advancing PTSD research and therapeutic development.

### 6.2. Genetic-Based Approaches

Genetic models have significantly advanced our understanding of the neurobiological underpinnings of PTSD, revealing intricate genetic factors that contribute to the disorder’s complexity. Moreover, the availability of genetically modified models for studying PTSD in rats has expanded considerably over the last few decades. Rat genome mapping and advancements in genomics have facilitated numerous studies aimed at identifying causal disease genes through positional identification. Over 350 rat genes have now been implicated in diseases or critical biological processes altered in pathological conditions, providing a rich resource of genetically engineered disease models [236,237]. The availability of genome-editing technologies, including gene-specific nucleases, has accelerated the development of targeted rat models in recent years [236,237].

Unlike traditional mouse models, rat models offer advantages such as better physiological similarity to humans, particularly in behavioral and neurobiological responses. For example, SERT(−/−) knockout rats display depression/anxiety-like behaviors and drug addiction-like tendencies, offering insights into the common dimensions of mental disorders, such as anxiety, depression, and drug addiction, which overlap with PTSD [238]. Co-morbid depression and PTSD, often resistant to treatment, have been effectively studied using male Flinders sensitive line (FSL) rats, a genetic model of depression. These rats exhibit bio-behavioral characteristics of depression, making them a suitable model for treatment-resistant depression in PTSD [123,239,240].

Genetic rat models have proven effective in mimicking PTSD-like behaviors, including impaired fear extinction, heightened anxiety, and despair-like symptoms. For example, selective breeding of Wistar Kyoto (WKY) rats has produced nearly isogenic substrains, such as WKY More Immobile (WMI) and WKY Less Immobile (WLI), which display divergent fear memory responses. WMI rats exhibit depression-like behaviors, while WLI rats do not, highlighting strain-specific differences in PTSD and depression susceptibility [241]. Knockout models targeting norepinephrine system enzymes (e.g., MAO-A, COMT) and genetic modifications of 5-HT receptors further clarify the role of these systems in regulating mood, anxiety, and fear responses [237,242,243,244]. Similarly, genetic modifications of the GABA_A_ receptor subunits underscore the importance of the GABA/benzodiazepine receptor complex in maintaining anxiety regulation [244].

Claude Szpirer (2020) summarized several genetically engineered rat models that have been instrumental in advancing our understanding of behavioral traits relevant to PTSD [237]. Among these are models exploring anxiety, depression, fear memory, and stress responses. For example, the Slc6a4 knockout rat exhibits anxiety and depression-like behaviors alongside altered DNA methylation in the urocortin promoter, while the Nr3c1 knockout mutant reveals sex-specific deficits in fear memory acquisition and extinction, paralleling coping behaviors during stress [237]. Similarly, the Crebbp and Rin1 knockouts display impairments in fear memory and extinction learning, making them valuable tools for studying PTSD-related traits [237]. Moreover, the Nrg1 knockout rat shows alterations in HPA axis activity and stress responses, directly linking genetic mutations with physiological and behavioral outcomes relevant to PTSD [237].

These advancements provide genetic models with strong construct validity by linking molecular and cellular findings to PTSD-related neurobiological mechanisms. For instance, corticotropin-releasing hormone (CRH) overexpression and CRH receptor knockouts delineate the distinct roles of these receptors in mediating anxiety and stress responses [237,244]. Transgenic rat strains with altered glucocorticoid expression have shed light on the neuroendocrine shifts associated with PTSD and depression, emphasizing the interplay between genetic predispositions and environmental stress [245].

While genetic models provide invaluable tools for unraveling the genetic and molecular underpinnings of PTSD, they face limitations, including challenges in fully replicating the human condition and the complex interplay between genetic and environmental factors. To address these limitations, combining genetic models with environmental and developmental paradigms, such as early-life stress exposure or social instability, can better elucidate gene–environment interactions that influence PTSD susceptibility and resilience. Such integrative approaches, including testing buffering factors like enriched environments or structured social support, offer a comprehensive understanding of PTSD’s multifaceted nature and potential therapeutic strategies.

## 7. Current Questions for the PTSD Research

### 7.1. Animal Model Resemblance to Human PTSD

In the study of PTSD, animal models are crucial for bridging the gap between theoretical research and clinical practice. These models are evaluated based on their phenomenological resemblance to human PTSD symptoms (face validity), their foundation in a logical theory or cause of the disorder (construct validity), and their ability to predict responses to treatment (predictive validity). The criteria proposed by Yehuda and Antelman enrich this validation framework by specifying five essential aspects, seamlessly blending with the three foundational concepts of validity [35]:

- Brief stressor induction: Demonstrates face validity by showing how short-term stressors can induce PTSD-like symptoms, mirroring the initial onset of the disorder.
- Intensity-dependent responses and persistence of alterations over time: Aligns with construct validity by illustrating that symptom severity increases with stressor intensity and that biological changes persist or become more pronounced over time, reinforcing the theoretical underpinnings of PTSD.
- Bi-directional expression: Enhances construct validity by acknowledging that symptoms can manifest differently under various conditions, adding depth to our understanding of the disorder’s complexity.
- Inter-individual variability: Supports predictive validity by recognizing that individual differences in genetics or past experiences can influence responses to treatment, mirroring the diverse reactions seen in humans.

There were attempts to characterize and categorize existing PTSD models according to the DSM-5 criteria for PTSD (see Table 6). Some models may satisfy virtually all criteria for PTSD (e.g., SPS, IS, and predator-based stress), while other models cover only some of them [17]. Certain models may even not be suitable to reproduce all important aspects of PTSD in humans; for example, housing instability has been shown to satisfy just five out of the eight main criteria of PTSD [17].

In addition to the variability between models, there is a key aspect of stress induction related to the complexity of social development and interaction. A critical difference between animal models and human PTSD is that humans can be profoundly stressed not only by direct trauma or injury but also by witnessing traumatic events happening to others [246,247]. It remains unclear whether animals are capable of experiencing more complex emotions, such as grief or a heightened state of horror when witnessing the death or injury of their companions. This adds another layer of complexity in choosing appropriate PTSD models, as these deeper emotional responses may be unique to human social behavior.

### 7.2. Sensitive Periods, Gender, and Memory

The timing of trauma exposure plays a pivotal role in the development and severity of PTSD symptoms, as well as their neural underpinnings. Studies utilizing animal models of fear conditioning have demonstrated significant age-related effects on fear learning, highlighting critical developmental windows for PTSD vulnerability. Middle childhood, approximately age 10 in humans (periadolescent period: PND 28–42 in rats), is particularly sensitive due to the ongoing development of the amygdala and its connectivity with the ventromedial prefrontal cortex. Trauma during this period can lead to outcomes such as heightened amygdala reactivity and volume, as observed in previously institutionalized children [248].

The brain’s sensitivity to environmental influences during early life extends to both adverse and protective factors. Early-life stress, such as prenatal chronic stress or disrupted rearing environments, significantly contributes to PTSD vulnerability in adulthood. Experimental paradigms inducing early-life stress include unpredictable footshocks during pregnancy [249], limited nesting/bedding for newborns (PND 2–21) [250], and maternal separation, where pups are separated from their mothers for 1–3 h daily (PND 2–9). These models consistently report depressive-like behaviors, impaired hippocampal-dependent spatial memory, and reduced long-term potentiation in the hippocampus during young adulthood (PND 53–57) [251,252]. Importantly, maternal separation models also exhibit sex-dependent changes in anxiety behaviors, acoustic startle responses, and HPA axis function, underscoring the role of sex as a modifier in early-life stress outcomes [253]. However, these paradigms are not PTSD-specific and are widely used to model other psychiatric disorders, such as anxiety, depression, and schizophrenia. This overlap complicates the interpretation of results, as the observed behavioral and physiological changes may reflect broader stress-related pathologies rather than PTSD alone.

Environmental factors during development can also buffer against early-life stress. Interventions like environmental enrichment during critical developmental periods (PND 22–52) have been shown to mitigate the adverse effects of prenatal stress and maternal separation on stress responses. For example, environmental enrichment during peripubertal periods reversed the endocrine and behavioral consequences of maternal separation [254,255,256]. While environmental enrichment does not prevent the immediate effects of stress exposure, it demonstrates the capacity to promote resilience and recovery.

Gender differences further influence PTSD susceptibility and its manifestations [105,106,107,108,109,198,199,200,201,202,255,256,257,258,259,260,261,262]. Women are nearly twice as likely as men to develop PTSD and mood disorders [255,256,257,258,259,260]. Between ages 21 and 25, women exhibit a threefold increased prevalence of PTSD compared to men [255], despite experiencing fewer traumatic events (77% likelihood compared to men) [260]. This heightened vulnerability is attributed to hormonal influences, sex-specific memory mechanisms, and differences in emotion regulation. Sex-specific studies have revealed notable differences in fear memory processes. For example, Riccardi et al. (2024) identified distinct male and female patterns in fear memory expression and extinction [12]. Ovariectomized female rats given estrogen replacement during stress exposure demonstrated enhanced dendritic remodeling in the prefrontal–amygdala inhibitory pathway, a change absent in males [262,263]. Similarly, in humans, women with PTSD exhibit reduced connectivity between the amygdala and the subgenual anterior cingulate cortex, impairing emotion regulation [264]. Sex differences also extend to autobiographical memory systems. Women tend to outperform men in episodic autobiographical memory tasks, excelling in verbal and sensory cues, while men show advantages in spatially demanding tasks [265,266,267,268,269]. Women’s greater activity in regions such as the dorsal anterior cingulate cortex and precentral gyrus during episodic autobiographical memory retrieval may explain this difference. Neuroimaging studies further suggest that women recruit prefrontal regions more intensively during emotion regulation compared to men, who exhibit more efficient amygdala suppression [270,271]. These findings indicate that while women may exhibit faster memory retrieval and greater emotional richness, they also show heightened emotional reactivity, contributing to PTSD susceptibility [272,273,274,275,276,277].

Future PTSD models must integrate these sex-specific and developmental factors to improve their translational relevance. Incorporating hormonal manipulations, such as estrogen replacement, alongside memory and emotion regulation paradigms, can help elucidate sex-specific pathways in PTSD. Paradigms like the DCOC model further provide insights into disrupted contextual memory processing, which is central to PTSD pathology. By accounting for sensitive periods, environmental modifications, and gender differences, researchers can refine animal models to better mimic the complexity of PTSD and develop tailored therapeutic interventions for both men and women.

### 7.3. The Problem of Phenotyping PTSD

It is well known that PTSD develops only in part of the population of animals or humans [74,153,167,278]. Although the lifetime prevalence of severely stressful events in Western populations can be as high as 75–80%, the symptoms and severity of PTSD vary inter-individually, and only 10 to 20% of exposed people do develop PTSD [233,279] and between 2 and 10% of the general population lives with sustainable symptoms for years [279,280,281,282]. It is interesting that the economic level of a country’s development does not seem to be a factor in the rate of lifetime prevalence of PTSD [280,281,283]. The rate of maladaptive responses decreases significantly after the acute phase and stabilizes over time. In military personnel and combatants with past active involvement in military activity, the prevalence may range between a few percent and almost 30% [284,285,286] which still constitutes only one-third of the imposed “population”. Moreover, the same experience in the same environment may result in different stress responses and PTSD incidence depending on the nationality of the affected people [284]. Twin studies suggest a heritability rate for PTSD risk of about 30–40%, highlighting the significant role of genetic factors; the genetic predisposition is significantly higher in females vs. males [287,288]. This disparity emphasizes the importance of including both exposure to risk factors and individual profiling in animal models to more accurately represent human PTSD [289]. On the other hand, the estimated rate of PTSD prevalence has also been shown to be dependent on the estimation approach (like the content of a survey) even if applied to the same population sample [290], which supports that the diagnostics and further classification of PTSD states is at least in part an arbitrary process.

Similar to the individual and gender-specific susceptibility to stress, the treatment of PTSD is not successful in a sized proportion of affected people, and this rate is dependent on the type, strength, and duration of stress—it can be termed as “individual resistance to treatment”. The individual resistance to PTSD treatment in the general population may vary between one-third and two-thirds depending on the treatment modality used, including psychological therapy and pharmaceutical treatment [291]. In military veterans, who likely suffer from the most severe stress, only approximately one-third display full remission after PTSD treatment therapy, but 72.8% of the remitted still would be qualified at least for one PTSD criteria [292]. In addition, the treatment recommendations do not differ for those in the acute state (symptoms persisting within a month) and in the chronic long-lasting state (symptomatic for many years) [293].

It is not surprising therefore that the efficacy of a certain treatment (either pharmacological or psychological, or both) for PTSD may work well in some patients and may be inadequate in others. More generally, different treatments may be required for different patients even if they were initially scored as having the same severity and symptoms of PTSD. The idea of developing a robust classification approach has emerged during the last two decades and advanced significantly in the last decade. Such a classification system aimed to elaborate standardized treatment guidelines for PTSD for giving specific treatment recommendations based on the characteristics of the disorder. Recently, a model of four-stage PTSD was proposed, where the PTSD severity ranges from a trauma-exposed asymptomatic but risk-prone state to severe unremitting chronically progressed illness [293]. This model utilizes various chronological characteristics of the disorder based on neurobiological markers, information processing systems, stress reactivity, and consciousness dimensions. The authors consider the model as the neurobiologically driven trajectory-based typology of PTSD and underline their utility for personalized recommendations for treatment interventions [293].

Recent research has increasingly focused on developing animal models that classify individuals as either resilient or susceptible to stress, thereby enhancing our understanding of the variability in PTSD-like symptoms. It has been demonstrated that animals can be categorized based on their individual responses to stressors, which aids in identifying neurobiological markers associated with resilience or susceptibility to stress [294]. Behavioral assessments have also been employed to classify rodents as resilient or susceptible, facilitating targeted exploration of the neural mechanisms underlying these divergent stress responses [81,295]. Significant behavioral and neuroendocrine differences have been observed between resilient and susceptible groups following repeated stress exposure, highlighting the value of such phenotyping in PTSD research [71,72,73,74,116,120,152,153,154,155,168,169,214,296]. Moreover, it was found that metabolic peculiarities could also predict PTSD susceptibility in rats [214]. These models are instrumental in advancing our understanding of PTSD and in developing personalized intervention strategies [214,294,295,296].

Other recent advances in this very important aspect of PTSD research include the development of EEG-based classification of PTSD symptoms by their severity as well as revealing the propensity to stress resilience in animals and in humans [297,298,299,300]. In addition, various diagnostic interview- and self-reporting-based classifications have been developed and validated through a clinician-administered PTSD scale to discriminate between measures of anxiety, depression, somatization, and functional impairment [301,302,303,304], as well as to adhere to the PTSD Checklist developed earlier [305]. However, the classification of distinct responses to stress and separation of the responders by PTSD phenotypes still needs further elaboration and, perhaps, nontrivial approaches. For instance, in one of the above-mentioned studies, researchers sought to simplify the classification of heterogeneous responses to stress among patients diagnosed with lifetime PTSD [302]. The study used Latent Class Analysis (LCA) to identify four distinct PTSD symptom profiles despite all participants meeting the DSM-5 criteria and PTSD Checklist requirements. These profiles were classified as dysphoric (23.8%), threat-reactive (26.1%), high symptom (33.7%), and low symptom (16.3%) [302]. This classification aims to reduce the complexity of PTSD symptom variability and improve treatment personalization.

Finally, it is worth noting two other psychological states in humans that may affect and/or shadow the direct consequences of PTSD and ameliorate or complicate the consequences. People who survived a trauma often develop psychological and neurobiological processes targeted at the rethinking of stressful events—this must not be interpreted as trauma re-experience, which is a symptom of PTSD itself. Generally, positive rethinking is called post-traumatic growth (PTG), and the generally negative one is known as post-traumatic depreciation (PTD); both are complex constructs involving rumination and core beliefs [306,307] and possibly deterministic thinking and/or the concept of destiny. In patients with proven PTSD symptoms, it has been demonstrated that PTG can still develop but not in all patients [308]. This finding highlights an even more complicated interrelation that may exist between the excitatory and inhibitory components of CNS. The extent of how this interrelation is unbalanced may not be a simply defined measure (and, importantly, it likely has a dynamic multifactor-dependent nature), but it greatly contributes to the individuality of PTSD manifestation, persistence, and resistance to the treatment.

### 7.4. From Adaptive Mechanisms to Pathological Outcomes

PTSD is a stress-triggered dysfunction of highly conserved brain systems involved in the regulation of anxiety, fear, and reward. The systems are the prefrontal complex (PFC), amygdala, and hippocampus, and many of the studies were performed using rodents and humans [309]. If functioning properly before stress, their function cannot be easily switched to the pathological state. The dichotomy between physiological responses and pathological outcomes, especially in the context of stress and trauma, presents a nuanced challenge in understanding the mechanisms underlying PTSD and other stress-related disorders. A perspective offered by Richter-Levin and colleagues [198], supported by research into early-life stress and predator stress models, suggests that many of the physiological, structural, and molecular changes observed following trauma may initially serve adaptive and resilience-building purposes [232,300]. This discussion revolves around the idea that some responses, traditionally viewed as pathological, might actually represent accelerated physiological processes. The dual nature of stress-induced physiological responses is highlighted in Table 7.

The dichotomy between physiological responses and pathological outcomes, particularly in the context of stress and trauma, presents a nuanced challenge in understanding the mechanisms underlying PTSD and other stress-related disorders. A perspective offered by Richter-Levin, Stork, and Schmidt (2019) suggests that many of the physiological, structural, and molecular changes observed following trauma may initially serve adaptive and resilience-building purposes [233]. This perspective is further supported by research into early-life stress and predator stress models, indicating that some responses, traditionally viewed as pathological, might actually represent accelerated physiological processes rather than inherently pathological states [233,310].

Maternal deprivation, for example, can be seen as an acceleration of the natural process of leaving the nest. In animal models, this is not necessarily pathological but rather a preparation for independent life, albeit brought about prematurely. This accelerated transition, while potentially creating a vulnerable phenotype, does not in itself constitute a pathological impact but rather a heightened state of preparedness for future stressors [310].

Similarly, classical fear conditioning, a fundamental learning process essential for survival, teaches animals to avoid or escape dangerous situations. The application of this model in PTSD research operates under the assumption that the mechanisms underpinning PTSD are analogous to those of classical fear conditioning, albeit intensified. However, PTSD may also arise from a failure of normal fear responses to severe trauma, suggesting the collapse of these adaptive mechanisms and the emergence of a pathological process. This distinction underscores the fine line between physiological adaptation and pathology when confronted with extreme stress.

The concept of the “training reaction” highlights a physiological response to stress that does not overwhelm the body’s protective systems but instead induces a mild anti-inflammatory effect and prepares the organism for future challenges [310]. The “training reaction” is characterized by increased secretion of glucocorticoids within the upper half of the normal range, promoting resilience without leading to immunosuppression. Following the “training reaction”, exposure to potentially traumatic stress may trigger an “activation reaction”, a nonspecific adaptive response to medium stimuli, including olfactory stress. This biological reaction aims to bolster the activity of the body’s regulatory and protective systems, featuring moderate excitation in the central nervous system and a balanced secretion of glucocorticoids. Such reactions serve the biological purpose of adapting to and overcoming immediate challenges while potentially setting the stage for stress-related disorders if the balance between adaptive responses and pathological processes is disrupted.

Exploring the realm of potentially non-pathological effects in the context of stress and trauma research requires a nuanced understanding of physiological responses that, although may seem maladaptive or harmful at first glance, play crucial roles in adaptation and survival. This reconsideration can lead to valuable insights into experimental models and their interpretations.

## 8. Conclusions

The current review provides a detailed analysis of the strengths and limitations of various rat models employed in PTSD research, including SPS, S-R, and predator-based paradigms, among others. While these models offer valuable insights into the neuroendocrine, behavioral, and genetic mechanisms underpinning PTSD, significant concerns about their reproducibility and the rigors of study design remain unresolved. This issue has been underscored by several prominent researchers and regulatory bodies, such as the NIH and leading scientific journal editors, who have emphasized the necessity of transparent reporting and rigorous methodological standards in preclinical research [310].

The lack of reproducibility is partly due to methodological variability, which includes differences in how these stress models are implemented across laboratories. To mitigate this, future research should adopt standardized protocols and detailed reporting [310]. Moreover, animal models should better incorporate the etiology of PTSD, which involves complex, interacting factors such as genetics, early-life stress, individual coping abilities, and social support mechanisms. Acute or chronic stress exposure alone, as induced in these models, does not fully capture the intricacies of PTSD development in humans, where susceptibility is influenced by a combination of genetic, environmental, and psychological factors. Thus, improving model validity requires incorporating individual variability and developing paradigms that include both the precipitating trauma and the subsequent risk-modifying influences.

These models must meet stringent validity criteria—face validity, construct validity, and predictive validity—to simulate effectively human PTSD. Despite advancements, achieving a perfect alignment with these criteria remains challenging. Models like SPS, S-R, TDS, and PSS have shown promise but often fall short in addressing variable symptom expression and capturing the complete spectrum of human PTSD. Pharmacological validation is crucial yet fraught with challenges due to the complexity of PTSD symptoms and inconsistent drug effects across species.

Based on the evaluation presented in Table 8, it is evident that different models have specific strengths suited to distinct research needs. For example, the SPS model provides consistent PTSD phenotypes and is standardized, making it suitable for exploring fear extinction associations, though it suffers from low reproducibility. Predator-based models offer high ethological validity and produce robust PTSD phenotypes, making them particularly useful for studies focusing on the genetic predisposition to PTSD. The S-R model, which simulates the PTSD trigger–response mechanism and responds to known treatments, could be valuable for examining HPA axis dysfunction and chronic stress effects. However, challenges with limited direct fear measures remain. For more targeted studies, Immobilization Stress Models provide extensive HPA axis data with insights into both male and female responses.

Ultimately, the choice of model should be driven by the specific research question—whether it is focused on neuroendocrine dynamics, behavioral phenotypes, or pharmacological responses. There is no single “best” model, but rather a selection of models each tailored to capture different aspects of PTSD. In conclusion, while animal models continue to be indispensable for advancing our understanding of PTSD, there is a pressing need for further refinements that enhance reproducibility, etiological validity, and translational applicability. A focus on rigorous study designs and more ecologically valid stress paradigms will improve the reliability of preclinical findings, ultimately supporting better translation from animal studies to clinical applications [17,310,311].

## 9. Recommendations for Ideal Model and Study Design in PTSD Research

The proposed “ideal” experimental design aims to develop a comprehensive rat model of PTSD that incorporates various stress exposures across different developmental stages to study sex differences, memory mechanisms, and pharmacological interventions. The experiment should involve key developmental stages: early life (postnatal day (PND) 2–21), adolescence (PND 28–42), and adulthood (PND 60 onwards).

While the experimental groups should include male rats, female rats, ovariectomized female rats with estrogen replacement, and ovariectomized female rats without estrogen replacement, each consisting of 20 animals, a large number of animals is necessary to further subdivide them into resilient and susceptible phenotypes. Additionally, control groups without social deprivation/separation for each of these categories should also be included, bringing the total number of animals to about 160. However, it is important to note that the exact number of animals required for the proposed design will ultimately depend on the specific analyses being conducted. The integration of different types of assessments—such as corticosterone level measurement, pharmacological interventions, and dendritic remodeling analysis—may require different sacrifice methods and endpoints, which could impact the number of animals needed per group. Therefore, flexibility in the experimental design must be maintained to ensure adequate sample sizes for each type of measurement, and adjustments should be made based on the specific methods and endpoints involved.

**Figure 5:**
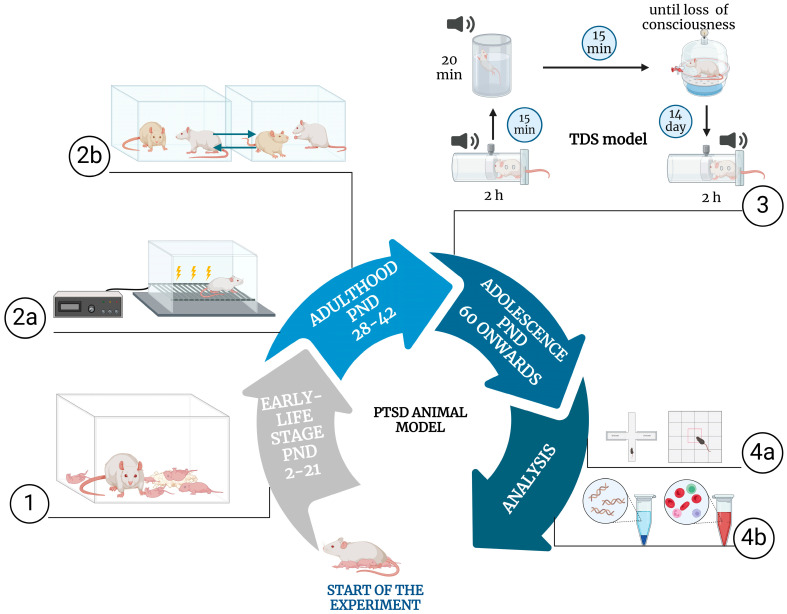
A progression plot for the proposed “ideal” design and strategy for experimental research using PTSD animal models (rats in this case).

Early-life stress should be induced by housing newborn rats in cages with limited nesting and bedding materials from PND 2 to PND 21 (Figure 5, step 1). In adolescence (PND 28–42), unpredictable mild footshocks should be administered (Figure 5, step 2a), and social instability should be created by changing cage mates daily (Figure 5, step 2b). In adulthood (PND 60 onwards), the rats should be subjected to a modified TDS model (Figure 5, step 3). The modification involves adding contextual auditory cues during stress exposures. These cues reinforce the traumatic experience and will later serve as triggers characteristic of PTSD.

Behavioral and neurobiological assessments should be conducted using the differential contextual odor conditioning (DCOC) paradigm, where rats are trained and tested with cinnamon odor in aversive, safe, and neutral environments to assess contextual memory modulation. Memory tasks, including the Morris water maze and novel object recognition test, should be used to evaluate episodic and semantic memory. Anxiety and stress responses should be measured using the elevated plus maze and open field tests, alongside corticosterone level assessments to evaluate HPA axis responses. Neurobiological assessments should include examining dendritic remodeling in the prefrontal cortex and amygdala (Figure 5, step 4a). Pharmacological interventions should involve administering paroxetine to evaluate its impact on PTSD-like symptoms and exploring the efficacy of ketamine treatment in alleviating these symptoms. Behavioral, neurobiological, biochemical, and molecular–genetic assessments should be conducted immediately after stress exposure and at 1 month and 3 months post-stress to observe long-term effects and treatment outcomes (Figure 5, step 4b).

The proposed experimental design aims to provide a comprehensive understanding of PTSD by integrating developmental timing, sex differences, memory mechanisms, adaptive versus pathological stress responses, and pharmacological validation. This type of study should create a more accurate and effective rat model that reflects the complexity of PTSD and facilitates the development of targeted interventions and therapies for both men and women.

In summary, refining animal models, enhancing pharmacological validation, understanding the balance between adaptive and pathological stress responses, and considering sensitive periods and gender differences will pave the way for more effective interventions and treatments for PTSD.

Note: Step 1 (Early-Life Stress: PND 2–21): Newborn rats are housed in cages with limited nesting and bedding materials, simulating early-life adversity. This phase models stress exposure during early developmental stages to observe its effect on later behavior and neurobiology. Potential interventions, such as enriched bedding, could also be tested to evaluate buffering effects.

Step 2 (Adolescence: PND 28–42)—Step 2a: Unpredictable mild footshocks are administered to induce stress, replicating unpredictable stressors experienced during adolescence. Step 2b: Social instability is induced by changing cage mates daily, creating fluctuating social conditions to mimic instability and its influence on stress response development. This phase could also incorporate stress-coping tasks and models of social interaction or isolation to explore resilience and vulnerability mechanisms.

Step 3 (Adulthood: PND 60 onwards): Rats are exposed to a modified Three-Day Stress (TDS) model, incorporating contextual auditory cues during stress exposures. These cues simulate PTSD triggers, modeling the persistence and re-experiencing aspects of PTSD in adulthood. Social support mechanisms could be introduced to evaluate their protective effects during this phase.

Step 4a and 4b (Behavioral and Neurobiological Assessments): Behavioral assessments, such as differential contextual odor conditioning (DCOC), the Morris water maze, and novel object recognition, evaluate the effects of stress on various types of memory. Anxiety and stress responses are measured through tests like the elevated plus maze and open field tests, with corticosterone levels assessed to monitor HPA axis reactivity. Neurobiological and pharmacological evaluations, including dendritic remodeling analysis and pharmacological interventions (e.g., paroxetine and ketamine), are conducted across multiple time points to observe both short-term and long-term effects.

Additional Considerations: Genetic Predispositions: Across all phases, genetic studies could be integrated, such as using genetically modified rat lines, to investigate hereditary factors in PTSD susceptibility.

Buffering Factors: Social and environmental interventions, such as enriched environments or structured challenges, could be explored to assess their role in mitigating stress-induced outcomes.

## Figures and Tables

**Table 1:** Spectrum of PTSD animal models: from single-stressors to complex multi-stressor approaches with temporal dynamics.

Model	Type of Stressor	Outcome	Stressor Application	Ethological Validity *	Etiological Validity *	Complexity
Electric Shock Models	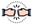 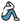	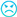 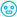 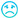 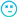	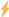 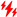	●	●●●	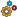
Immobilization Stress	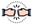	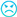 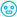	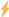 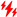	●	●●	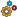
Underwater Trauma and Water-Associated Zero Maze	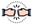	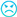 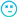	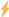 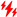	●●	●●●	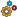
Single Prolonged Stress	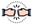 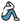	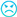 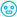 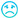 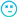	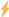 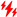	●●	●●	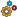
Unpredictable Variable Stress	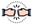 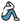	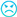 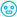 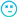	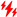	●●	●●●	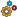
Stress–Re-stress	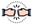 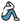	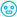 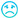	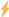 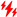	●●	●●	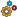
Time-Dependent Sensitization	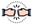 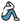	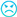 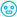 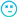	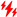	●●	●●●	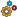
Acoustic Startle Response	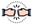 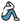	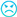 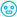	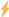	●	●●	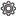
Social Stress Models	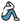	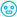 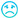	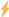 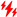	●●	●●	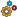
Predator-Based Models	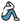	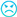 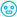	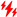	●●●	●●●	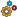
Pharmacological Models	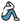	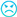 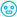 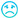	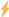	N/A	●	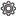
Genetic Models	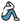	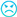 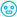 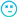	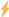 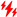	N/A	●●	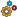
Differential Contextual Odor Conditioning	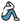	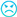 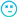	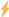 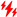	●●	●●	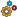

Notes—Visual Symbols: 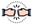
—physical Stressor; 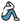
—non-physical stressor; 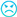
—anxiety; 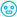
—fear; 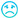
—depression; 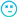
—cognitive impairment; 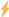
—single stressor; 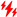
—multiple stressors; ●●●—high ethological or etiological validity; ●●—medium ethological or etiological validity; ●—low ethological or etiological validity; 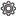
—simple model; 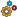
—complex model. * Ethological validity refers to how well the behavior of an animal model reflects natural behaviors or human-like conditions, focusing on observable phenotypes relevant to both species. In contrast, etiological validity concerns whether the causes of a disease or condition in the animal model mirror those in humans, emphasizing the underlying mechanisms leading to the disease. In the context of pharmacological and genetic PTSD Models, ethological validity may not be directly applicable due to their mechanistic focus (indicated as “N/A”—not applicable).

**Table 2:** Comparison of single prolonged stress (SPS), stress–re-stress (S-R), and time-dependent sensitization (TDS) models in PTSD research.

Model	Type of Stressor	Components	Targeted Symptoms	Key Findings	Strengths	Specific Weaknesses
SPS	Multi-Stressor	Restraint, forced swim, ether anesthesia	Acute stress response, hyperarousal	HPA axis dysregulation, impaired fear extinction	Widely used, models acute stress effects effectively	Lacks representation of chronic symptoms
S-R	Multi-Stressor	Restraint, forced swim, ether, re-stress	Chronic aspects of PTSD, anxiety	Anxiety-like states, HPA axis dysfunction	Models chronic stress aspects and situational reminders, effective in inducing HPA axis dysregulation	Limited capacity to simulate hyperarousal symptoms
TDS	Multi-Stressor and Ethological Validity	Restraint, forced swim, ether, situational reminder	Situational reminders, sustained PTSD symptoms	HPA axis dysregulation, prolonged stress effects	Ethologically valid, captures both acute and chronic stress effects, incorporates reminders	Limited symptom expression, requires further physiological and behavioral measures
General Weaknesses	Low reproducibility, methodological variability, lack of standardization in scoring, absence of randomization, inadequate sample size calculations. High variability in outcomes, difficulty in conducting meta-analyses.					

**Table 3:** Pros, cons, and limitations of social stress models in PTSD research.

Model	Description	Strengths	Weaknesses	References
Social Isolation	Prolonged isolation leading to stress-induced behavioral and physiological changes.	Models aspects of social withdrawal and anxiety-like behavior; Reproducibility.	Limited ethological relevance; Lack of specificity, overlap with symptoms of depression or anxiety; Difficult generalization to human PTSD.	[134,135,136,137,138,139,140]
Housing Instability	Instability in housing environment leading to chronic stress.	Mimics real-world environmental instability; Assesses combined physical and psychological stress.	Highly variable outcomes depending on setup; Results may not generalize well.	[135,136,137,138]
Juvenile Social Exploration	Introduction of a juvenile rat to assess social behavior and anxiety response.	Provides insights into social avoidance and resilience mechanisms; Relevance to stress-induced analgesia and learned helplessness.	Validation challenges in prolonged stress incubation models and conspecific exploration.	[144,145,146]
Social Defeat Stress	Introduction of intruder into the resident’s territory to induce a defeat episode.	Models PTSD-like symptoms such as heightened fear and anxiety; Stable and reproducible outcomes; Differentiates between resilient and susceptible individuals.	Lacks protocol for social stress in females; Not suitable for single traumatic exposure modeling.	[138,139,140,141]

Notes—Social isolation (SI): Typically demonstrates behaviors like increased aggression or anxiety, but these may not always align with PTSD, making it difficult to discern underlying causes accurately. Variables such as age at isolation and genetic factors affect the generalizability of SI models. Housing instability (HI): HI models are valuable in replicating stress due to environmental instability but can produce varied results based on the specific parameters used. Juvenile social exploration (JSE): JSE particularly enriches understanding of learned helplessness, but experimental consistency and validation remain challenging. Social defeat stress (SDS): SDS effectively mimics social-induced PTSD-like symptoms and allows clear differentiation between resilient and susceptible individuals; however, female-specific models remain underdeveloped.

**Table 4:** Pros, cons, and limitations of predator-based models in PTSD research.

Aspect	Details	References
Pros		
Ethological Validity	Uses naturalistic threats, closely mirroring real-life stressors.	[22,182]
Absence of Physical Harm	Focuses on psychological stress without causing physical injuries.	[182]
Sensitivity to Treatment	Produces phenotypes responsive to treatments like selective serotonin reuptake inhibitors (SSRIs).	[15]
Versatile Stimuli	Allows the use of natural and synthetic stimuli.	[173,175,176]
Reveals Genetic Predisposition	Identifies animals susceptible or resilient to PTSD-like conditions.	[15,183,191]
Induces Long-Term Effects	Provokes enduring PTSD-like (patho)physiological and behavioral responses.	[151,182]
Cons and Limitations		
Variability in Responses	Not all animals develop PTSD-like symptoms, complicating reproducibility.	[183,184,196]
Limited Research in Females	Limited focus on females, especially in areas like sleep disturbances and depressive behaviors.	[199,202]
Complicating Protocols	Variability in protocols and rodent strains complicates findings; secondary stressors may be needed.	[183,196,200]
Limited Scope of PTSD Symptoms	Does not fully account for episodic memory alterations and memory dissociation.	[201]
Synthetic Stimuli Limitations	Synthetic odors like TMT do not fully replicate the effects of natural predator odors.	[173,177,178,180,181]

**Table 5:** The pharmacological models of various PTSD-like states.

Model Type	Examples	Action	Key Features	References
Glucocorticoid Hormone Synthesis Inhibition	Metyrapone	Inhibits 11β-hydroxylase; affects HPA	Reduces glucocorticoid levels, influencing anxiety and cognitive functions.	[97,217,218,219]
Corticosterone Administration	Corticosterone	Modulates anxiety-like behavior	Mimics chronic stress conditions; prevents anxiety-like behavior induced by immobilization stress.	[155,220,221]
CRF Administration	CRF injections	Elevates stress response	Induces anxiety-like behaviors; disrupts HPA axis function.	[222,223,224]
Anxiogenic Agents	FG-7142	GABA_A_ receptor inverse agonist	Induces anxiety and hypervigilance; useful for studying GABAergic mechanisms.	[225,226,227,228,229]
Adrenergic Challenge	Yohimbine	Alpha-2 adrenergic receptor antagonist	Provokes hyperarousal and anxiety by enhancing noradrenaline release.	[227,228,229]
Ecstasy-like Compounds	MDMA	Neurotoxicity at high or repeated doses	Alters mood, perception, and social behavior; studied for neurotoxic effects.	[230]
5-HT2A Inverse Agonism	Pimavanserin	5-HT2A inverse agonist	Reverses persistent stress effects in stressed rats.	[231]

Note: CRF—corticotropin-releasing factor, MDMA—3,4-methylenedioxymethamphetamine.

**Table 6:** Validity and application of PTSD models in research.

Model	Face Validity	Construct Validity	Predictive Validity
Single Prolonged Stress (SPS)	Brief stressor induction Resemblance to human PTSD symptoms	Intensity-dependent responses Persistence of alterations	Inter-individual variability Response to known PTSD treatments
Stress–Re-stress (S-R)	Simulates trigger–response Resemblance to human PTSD symptoms	Outcome depends on re-stress treatment: - Footshock produces HPA axis dysfunction and prominent PTSD symptoms. - Forced swimming is less Effective.	Predicts treatment efficacy Response to known PTSD treatments
Time-Dependent Sensitization (TDS)	Captures acute and chronic stress Resemblance to PTSD symptoms	Corticosterone level changes Monoamine dysregulation	Predictive of therapeutic outcomes
Predator Scent Stress (PSS)	Naturalistic threats Resemblance to PTSD symptoms	Induces long-term HPA axis changes Neuroinflammation	Response to SSRIs and anti-inflammatory drugs
Social Defeat Stress (SDS)	Simulates chronic social stress Resemblance to PTSD symptoms	HPA axis impact Neurobiological changes	Predicts antidepressant efficacy
Early-Life Stress (ELS)	Models early trauma Resemblance to human PTSD symptoms	Long-term effects on brain and stress systems	Predicts mitigation effects of early stress treatments

**Table 7:** Dual nature of stress-induced physiological responses.

Physiological Response	Adaptive Effect	Potential Pathological Outcome
Stress-Induced Neurogenesis	Enhances learning and memory. Prepares the brain for future stressors by increasing resilience.	May lead to aberrant neural circuit formation. Overproduction of neurons could disrupt existing networks, leading to cognitive deficits or maladaptive behaviors.
Inflammation	Facilitates tissue repair and recovery. Clears cellular debris after injury.	Chronic inflammation can lead to tissue damage, fibrosis, and the development of diseases such as autoimmune disorders or chronic pain syndromes.
HPA Axis Hyperactivity	Mobilizes energy resources. Enhances immune function. Improves cognitive and emotional processing under acute stress.	Prolonged hyperactivity can result in impaired immune function, increased risk of metabolic disorders, and mental health issues like depression or anxiety.
Autonomic Nervous System (ANS) Reactivity	Prepares the organism for fight-or-flight responses. Enhances survival during immediate threats by increasing alertness and readiness.	Chronic hyperarousal can lead to cardiovascular diseases, anxiety disorders, and impaired stress coping mechanisms, contributing to a constant state of heightened anxiety or panic.

**Table 8:** Summary of PTSD models regarding their pros, cons, and corticosterone levels.

Model	Pros	Cons	Corticosterone Levels
Electric Shock Models	High control over stressor intensity and duration. Effective in simulating PTSD-like symptoms. Useful for fear conditioning.	Not ethologically valid. Potential physical harm. Inconsistent reflection of PTSD indicators.	Variable, often elevated acutely.
Immobilization Stress Models	Extensive HPA axis data. Reliable effects on fear-specific processes. Includes data on both sexes.	Potential physical injuries. Limited naturalistic relevance. Few long-term studies.	Elevated acutely, with increased feedback, especially in females.
Underwater Trauma and WAZM	Naturalistic stress without physical harm. Insights into hippocampal-dependent functions.	High stress intensity may vary. Ethical concerns. Potential injury risk in WAZM.	Decreased post-stress.
Single Prolonged Stress (SPS)	Consistent PTSD phenotypes. Standardized procedure. Reveals fear extinction associations.	Low reproducibility. Fails to elicit trauma-cue avoidance. Methodological variability.	Elevated, challenging PTSD specificity.
Unpredictable Variable Stress (UVS)	Valid for repeated traumatic events. Identifies susceptible/resilient groups.	Reproducibility challenges. Limited data on females. Inconsistent outcomes.	Enhanced negative feedback.
Stress–Re-stress (S-R)	Simulates PTSD trigger–response. Induces HPA axis dysfunction. Responds to known treatments.	Lacks direct fear measures. Confounds depressive-like behavior. Limited treatment effectiveness.	Hypocorticosterone observed.
Time-Dependent Sensitization (TDS)	Combines acute/chronic stress. Maintains stress responses with reminders.	Limited symptom expression. Requires more behavioral/physiological measures.	Variable.
Acoustic Startle Response (ASR)	Evaluates anxiety/hyperarousal. Useful for screening trauma susceptibility/resilience.	Predictive value varies with timing. Modulated by contextual factors.	Often acutely elevated.
Differential Contextual Odor Conditioning (DCOC)	Assesses contextual memory modulation. Explores cue-triggered memory and behavior. Relevant for PTSD models.	Requires precise control of environmental contexts. May not fully replicate complex PTSD symptoms.	Variable
Social Stress Models	Highlights the social environment’s impact. Provides insights into stress-induced changes.	Limited ethological relevance. Varies with social conditions/age. Difficult to control variables.	Variable; often elevated.
Predator-Based Models	High ethological validity. Produces robust PTSD phenotypes. Highlights genetic predisposition.	Response/protocol variability. Limited research on sleep disturbances. Differences in rodent strains.	Typically elevated.
Pharmacological Models	Targets specific neurochemical pathways. Provides insights into treatments.	Cannot fully replicate PTSD. Inconsistent effects across species.	Varies with the agent used.
Genetic Models	Explores genetic factors. Enables study of susceptibilities/treatment efficacy.	Limited by genetic modification specificity. May not capture full environmental interaction.	Varies widely with genetic modification and context.

## Data Availability

No new experimental data were created.
